# The transcription factor c-Jun inhibits RBM39 to reprogram pre-mRNA splicing during genotoxic stress

**DOI:** 10.1093/nar/gkac1130

**Published:** 2022-12-08

**Authors:** Florence Lemaitre, Fatima Chakrama, Tina O’Grady, Olivier Peulen, Gilles Rademaker, Adeline Deward, Benoit Chabot, Jacques Piette, Alain Colige, Charles Lambert, Franck Dequiedt, Yvette Habraken

**Affiliations:** Laboratory of Gene Expression and Cancer, GIGA-Molecular Biology of Diseases, B34, University of Liège, Liège 4000, Belgium; Laboratory of Virology and Immunology, GIGA-Molecular Biology of Diseases, B34, University of Liège, Liège 4000, Belgium; Laboratory of Gene Expression and Cancer, GIGA-Molecular Biology of Diseases, B34, University of Liège, Liège 4000, Belgium; Metastasis Research Laboratory, GIGA-Cancer, B23, University of Liège, Liège 4000, Belgium; Metastasis Research Laboratory, GIGA-Cancer, B23, University of Liège, Liège 4000, Belgium; Laboratory of Virology and Immunology, GIGA-Molecular Biology of Diseases, B34, University of Liège, Liège 4000, Belgium; Department of Microbiology and Infectious Diseases, Faculty of Medicine and Health Sciences. Université de Sherbrooke, Sherbrooke, Québec, Canada; Laboratory of Virology and Immunology, GIGA-Molecular Biology of Diseases, B34, University of Liège, Liège 4000, Belgium; Laboratory of Connective Tissues Biology, GIGA-Cancer, B23, University of Liège, Liège 4000, Belgium; Laboratory of Connective Tissues Biology, GIGA-Cancer, B23, University of Liège, Liège 4000, Belgium; Laboratory of Gene Expression and Cancer, GIGA-Molecular Biology of Diseases, B34, University of Liège, Liège 4000, Belgium; Laboratory of Gene Expression and Cancer, GIGA-Molecular Biology of Diseases, B34, University of Liège, Liège 4000, Belgium; Laboratory of Virology and Immunology, GIGA-Molecular Biology of Diseases, B34, University of Liège, Liège 4000, Belgium

## Abstract

Genotoxic agents, that are used in cancer therapy, elicit the reprogramming of the transcriptome of cancer cells. These changes reflect the cellular response to stress and underlie some of the mechanisms leading to drug resistance. Here, we profiled genome-wide changes in pre-mRNA splicing induced by cisplatin in breast cancer cells. Among the set of cisplatin-induced alternative splicing events we focused on COASY, a gene encoding a mitochondrial enzyme involved in coenzyme A biosynthesis. Treatment with cisplatin induces the production of a short isoform of COASY lacking exons 4 and 5, whose depletion impedes mitochondrial function and decreases sensitivity to cisplatin. We identified RBM39 as a major effector of the cisplatin-induced effect on COASY splicing. RBM39 also controls a genome-wide set of alternative splicing events partially overlapping with the cisplatin-mediated ones. Unexpectedly, inactivation of RBM39 in response to cisplatin involves its interaction with the AP-1 family transcription factor c-Jun that prevents RBM39 binding to pre-mRNA. Our findings therefore uncover a novel cisplatin-induced interaction between a splicing regulator and a transcription factor that has a global impact on alternative splicing and contributes to drug resistance.

## INTRODUCTION

Alternative splicing (AS) of pre-mRNA is a key molecular event in eukaryotic gene expression. By selecting which regions of the pre-mRNA are retained (exons) or removed (introns) to form the mature transcript, AS controls qualitative diversity of the transcriptome and expands the functional repertoire of the proteome. Pre-mRNA splicing is accomplished by the spliceosome, a large dynamic and complex molecular machine comprising 5 small nuclear RNAs and more than 300 proteins (1). AS takes advantage of the remarkable plasticity of the spliceosome in substrate recognition. The activity of the core spliceosome and the selection of specific parts of the pre-mRNA into the mature transcript are under the combinatorial influence of multiple splicing regulators. The identification of the complete set of splicing regulatory proteins that locally decides the exon/intron fate of any given RNA sequence remains one of the biggest challenges in the field. Historically, *trans*-acting RNA binding proteins (RBPs) that positively or negatively modulate the splicing process by binding to *cis*-regulatory sequence elements found in the pre-mRNA have been the best characterized (2,3). Recently, large-scale screens based on systematic gene inactivation have revealed that splicing regulation also involves factors classically associated with other regulatory layers of gene expression (4–6). Especially unexpected, DNA-binding proteins and transcription factors (TFs) were found to be over-represented among splicing regulators (4). However, in most cases, TFs impact pre-mRNA splicing indirectly, by controlling the expression of direct splicing regulators or RNA polymerase II elongation rate. In contrast, evidence for a direct, transcription-independent role of TFs in the regulation of AS remains scarce (7–9).

RNA binding motif protein 39 (RBM39, a.k.a CAPERα, HCC1, RNPC2) is an SR rich protein with two central RNA recognition motifs (RRM) and one C-terminal U2AF homology motif. Consensus on its specific RNA binding sequences has not yet been reached (10,11). In addition to RNA, RBM39 binds several proteins involved in spliceosome assembly, such as U2AF65 and SF3B1 but also U1-70K suggesting a possible role in splicing decisions, by positioning at both 3’ and 5’ splice sites (12). Its depletion leads to numerous splicing alterations (10–12). RBM39 is over-expressed in several types of cancer, *e.g*. breast and non-small cell lung cancers, colorectal adeno-carcinoma and acute myeloid leukemia among others and was recently implicated in sensitivity of cancerous cells to Indisulam, an anticancer sulfonamide (13–15). In addition to its splicing function, RBM39 was first characterized as a co-activator of activator protein-1 (AP-1) and estrogen receptor (ER, hence its name CAPERα) and later reported to affect the transcriptional activity of the steroid receptor and NF-κB (16–18). RBM39 controls AP-1 TF activity through direct binding of c-Jun, a major component of the dimeric AP-1, which is involved in cell proliferation, survival, apoptosis and tumour progression (19,20). The role of RBM39 in the control of AP-1 transactivating function is well documented but the potential role of c-Jun in the control of RBM39’s splicing function is not.

Treatment of cancer is still largely based on the use of chemotherapeutic drugs among which DNA-damaging agents such as cisplatin have held a prominent position for decades. Cisplatin, one of the most widely used anticancer drugs induces cell death through binding to genomic and mitochondrial DNA. It establishes inter- and intra-strand crosslinks affecting replication as well as transcription and leads to cell cycle arrest, apoptosis and ROS production by the mitochondria (21,22).

One of the main problems in chemotherapeutic treatments remains the acquisition of drug resistance by cancer cells along important side effects (23). Understanding the mechanisms of tumour resistance to genotoxic therapies is of crucial importance to develop novel drug regimens with increased therapeutic efficacy. Resistance to cisplatin is multifactorial. The involvement of mitochondria in this resistance is accepted but the precise molecular mechanism remains under-studied (24). Mitochondria are dynamic organelles whose shape and length result from an equilibrium between fusion and fission and vary throughout cell cycle and in response to stress (25,26). Upon cisplatin addition mitochondrial dynamics is modified and conversely mitochondria are involved in Cisplatin-induced cellular responses (22,24).

DNA-damaging agents trigger a complex network of responses called the DNA-damage response (DDR) that culminates in dramatic reprogramming of gene expression, which mostly relies on NF-κB and p53 and leads to the activation of DNA-damage repair and tolerance processes and cell-cycle checkpoint pathways (27,28). Recent transcriptomic profiling studies have revealed that in addition to changes in mRNA levels, DNA damaging agents promote large-scale modifications of the mRNA splicing landscape (29–32). As observed for changes in expression levels, changes in splicing mainly affect genes associated with DNA repair, cell-cycle control and apoptosis, but also transcriptional and post-transcriptional regulators, thus providing the opportunity to not only directly weigh on cell fate but also to feedback on every aspect of the DDR (33). How the DDR alters the specificity of the spliceosome to affect only specific AS events remains unclear but so far is thought to involve transcriptional activation and/or post-translational modification of RBPs by DDR signalling pathways (30,34,35).

Coenzyme A synthase (COASY), localised in mitochondrial membranes, carries-out the final two steps of the *de novo* biosynthesis of coenzyme A cofactor with its two catalytic domains: the 4’ -phosphopantetheine adenylyltransferase (PPAT) and the dephospho-CoA kinase (d-pCoAK) (36). Both steps are ATP dependent. Three different COASY isoforms have been described. The ubiquitously expressed α-isoform, the β-isoform bearing an additional N-terminal 29 AA and only expressed in the brain and the γ-isoform with only the d-pCoAK domain. COASY mutations lead to distinct neurodegenerations linked to mitochondrial dysfunction (37,38). Links with cancer have also been documented: (i) COASY forms a complex with p85alphaPI3K and affects PI3K signaling (39) (ii) it mediates radiation resistance via PI3K signaling in rectal cancer (40) and (iii) its depletion leads to hyper-acetylation of CPB and Aurora Kinase, elongating mitosis and favoring multi-nucleation in different cancer and non-cancerous cells (41).

In this study, we attempted to uncover the molecular mechanisms by which DNA damage impacts splicing decisions in cancer cells and the functional contribution of DNA damage-induced splicing changes to cellular sensitivity to genotoxic drugs. As a prototypical model to address these questions, we identified genome-wide changes in mRNA splicing induced by cisplatin in breast cancer cells and characterized the underlying regulatory pathways and the functional relevance of AS to drug sensitivity.

## MATERIALS AND METHODS

### Reagents and antibodies

Cisplatin [*cis*-diamineplatinum (II) dichloride (P4394)], camptothecin (C9911), doxorubicin (D1515), H_2_O_2_ (H1009) and etoposide (E1383) were purchased from Sigma-Aldrich (Saint-Louis, MO, USA). The stock solution of cisplatin (2.5 mM in water) was kept at –20°C and renewed every month. JNK-IN-8 (420150) was purchased from Calbiochem (San Diego, CA, USA) and MitoTracker Red CMXROS (9082) from Cell Signaling Technology (Danvers, MA, USA). RNAse A (740505) was from Macherey-Nagel (Allentown, PA, USA). Accutase (L0950-100) was from Biowest (Nuaillé, France). The list of primary and secondary antibodies used in this study is provided in Supplementary Table S1.

### Plasmids

p-DEST-Flag-RBM39, p-SPICA-N1-RBM39, p-SPICA-N2-c-Jun were built with the GATEWAY technology (Invitrogen, Carlsbad, CA, USA) starting from p-DONR223-RBM39 or p-DONR223-c-Jun obtained from the human ORFeome v5.1 (Center for Cancer system Biology of the Dana Farber Cancer Institute). The pcDNA-MYC-COASY-FL, kindly provided by Dr I.T. Gout (University College of London, UK), was used as a template to produce pcDNA-MYC-COASY-short lacking exons 4 and 5. Deletion of exons 4 and 5 of COASY was performed using Q5® Site-Directed Mutagenesis system (New England Biolabs, Ipswich, MA, USA). All constructs were verified by sequencing.

### Cell lines, transfection and transduction

For siRNA-based screening of RBPs, MCF-7 cells were grown in DMEM (Wisent Bioproducts, Saint-Bruno, Canada) supplemented with 10% FBS. Cells were transfected by siRNAs using Lipofectamine®2000 (Invitrogen) and total cellular RNAs were purified 72 h later. For all other experiments, MCF-7 cells were grown in DMEM (Lonza, Verviers, Belgium) and either transfected with siRNAs using Lipofectamine RNAiMax (Invitrogen) or transfected with expression plasmids using JET PEI (Polyplus, Strasbourg, France) according to the manufacturer's instructions. The siRNA sequences are available in Supplementary Table S1.

MEWO cells were cultured in McCoys media, HeLa in EMEM, U2OS, HEK293 and A549 in DMEM. THP1, A2780A/A2780-DDP and HCT8A/HCT8-DDP were cultivated in RPMI1640. All culture media were bought from Lonza and complemented with 10% FBS. Human Umbilical Vein Endothelial Cells (HUVEC) obtained from Lonza were cultivated in EGM™-2 (Lonza) with 2% FBS. Occasionally, the cisplatin resistant cell lines (A2780-DDP and HCT8-DDP) were challenged by exposure to 50 μM cisplatin for 1 h. The absence of mycoplasma contamination was assessed on a regular basis by RT-qPCR by the GIGA-viral vector platform (Liège, Belgium).

### RNA purification, end-point RT-PCR and RT-qPCR

For the siRNA-based screening of RBPs, total RNA was purified three days after siRNA transfection using TRIzol and quantified using the Lab-on-Chip station (Agilent Inc, Santa Clara, CA, USA). A total of 2 μg of RNA was reverse transcribed using a mix of random hexamers and oligo (dT) and Omniscript reverse transcriptase (Qiagen, Hilden, Germany). Twenty nanograms of cDNA were amplified with HotStarTaq DNA polymerase (Qiagen). Visualisation and analysis of amplified products were done using the LabChip HT DNA assay on an automated microfluidic station (Caliper Life Sciences, Hopkinton, MA, USA). For all other alternative splicing analyses, total RNA from fresh or frozen cells (–80°C) was isolated using the High Pure RNA isolation kit (Roche, Indianapolis, USA) or NucleoSpin RNA (Macherey-Nagel, Düren, Germany) and quantified by spectrophotometry (Nanodrop ND-1000, Isogene Life Science, De Meern; Netherland). Total RNA (20 ng) was reverse-transcribed, and cDNA amplified using Tth DNA polymerase (Roche). Primers were designed *in silico* (NCBI primer-blast) and selected within the exons upstream and downstream of the alternatively skipped exon(s) to simultaneously amplify long and short isoforms. Details for end-point PCR are described in Gabriel *et al.* (32) and in Supplementary Table S1. Amplicons were resolved by electrophoresis on polyacrylamide gel run in 0.5 TBE. After staining with GELSTAR dye (LO50535, Lonza), the signal was monitored using LAS4000 Biomolecular Imager (GE Healthcare Life Sciences, Chicago, IL, USA) and quantified with ImageQuantTL software (GE Healthcare Life Sciences). For analysis of quantitative gene expression by RT-qPCR, RNA purified as above was reverse transcribed using the RevertAid H Minus first strand cDNA Synthesis kit with random primers (Thermo Fisher, Waltham, MA, USA). The cDNA was next amplified using FastStart Universal SYBR Green Master (ROX) (Roche) or SYBR Green Master Mix Blue (Eurogentec, Seraing, Belgium) on a Light Cycler 480 (Roche). The efficiency of primers designed in silico (NCBI primer-blast) or found in literature was evaluated by the PCR standard curve method and their specificity assessed post-amplification by examination of the melting curve. Experiments were conducted a minimum of three independent times with each data point run in triplicate. Relative quantification of targets, normalized to an endogenous control (GAPDH), was performed using the comparative Delta delta Ct method. Results are relative mRNA levels compared to control conditions (mean ± SD). Primer lists for end-point PCR and RT-qPCR are provided in Supplementary Table S1.

### Next generation RNA sequencing and data processing

MCF-7 cells were transfected with control (*Ctl*) *siRNA, siRBM39#2* or *sic-Jun#1* in six-well plates. After 24 h the medium was changed, and cells were treated by cisplatin for 24 h or mock-treated. Total RNA was isolated using the High Pure RNA isolation kit and integrity was checked on an Agilent Technologies 2100 Bioanalyzer (RNA nano-chip). Three independent RNA preparations were used. All samples had an RNA integrity number value >8 and were processed with Truseq Stranded mRNA Sample Preparation kit from Illumina. Final library validation was done on Qiaxcel (Qiagen) to check library profile and by qPCR for quantification (Kapa Syber fast). Based on qPCR values, libraries were diluted and pooled in an appropriate loading concentration (1.2 nM). Sequencing was done on Novaseq 6000 (Illumina), paired end 2 × 150 bp (25% of 1 lane on Novaseq S4 flow cell / XP workflow).

Reads were mapped to the human GRCh38 transcriptome and quantified using Salmon v0.8.2 (42). Read counts were summed to the gene level using tximport v.1.2.0 (43). Differential expression of genes in cells transfected with c-Jun or RBM39 siRNA compared to *Ctl siRNA* and cisplatin-treated cells compared to untreated cells was assessed as pairwise comparisons with DESeq2 v.1.14.1 (44). Genes were considered to be differentially expressed if the adjusted *P*-value was less than 0.05. Differential splicing analysis in cells treated with *sic-Jun* or *siRBM39* compared to *Ctl* siRNA and cisplatin-treated compared to untreated cells was assessed as pairwise comparisons with rMATS 4.0.2 (45) using the Ensembl human genome annotation (GRCh38). Splicing events were quantified using both reads that span splice junctions and reads mapping to exons (JCEC). Splicing events were considered to be altered if false discovery rate (FDR) was <0.05.

Gene Ontology (GO) enrichment analysis was performed using DAVID 6.8 (46) on the set of differentially expressed (adjusted *P*-value < 0.05) protein-coding genes or the set of genes with significantly altered exon skipping events (FDR < 0.05).

Gene Set Enrichment Analysis was performed with GSEA 4.0.3 (47) using protein-coding genes ranked by DESeq2 log_2_FoldChange values, the classic (unweighted) enrichment statistic and the Hallmark gene or the C3 transcription factors sets. In addition, genes presenting cisplatin-induced AS were compared to C3 transcription factor MSigDB data sets using GSEA’s compute overlaps tool.

Downstream analyses were performed and plots generated using Microsoft Excel, GraphPad Prism 8.0 and R (48) and the R packages dplyr (48) and gene overlap.

### ChIP-Seq analysis

ChIP-seq data from the ENCODE project (ENCLB508LER) were re-analysed using the nf-core ChIP-seq pipeline v1.1.0. We used the *Homo sapiens* reference genome GRCh38. Integrative genomic viewer version 2.8.9 was used to visualise data.

### Immunofluorescence and mitochondrial dynamics

To study mitochondrial dynamics, cells growing on glass coverslips in 24-well plates were incubated with MitoTracker Red for 20 min at 37°C to stain the mitochondria. The cells were then washed with PBS, fixed 20 min in 4% paraformaldehyde-PBS at room temperature. When needed the cells were permeabilized in 0.2% Triton X100-PBS and blocked with 3% BSA/PBS then incubated 1 h with the primary antibody diluted in 3% BSA/PBS. After three washes in 0.1% Triton X100-PBS, the coverslips were incubated with Alexa 488 coupled secondary antibody for 30 min. After two washes, the nuclei were stained with Hoechst 33342 diluted 1/50 000 (Acros Organic, Gell, Belgium) and further washed twice with cold water, then the coverslips were mounted on glass slides with Mowiol® 4–88 (Sigma). Images were recorded with a Leica SP5 A0B5 confocal inverted microscope using a 21°C HCX PL APO CS 63 × glycerol objective or a Zeiss LSM 980 with a Plan Apochromat 63×/1.40 oil DIC f/ELYRA objective. The acquisition programs were LAS-AF or Zen-blue depending on the microscope used.

### Mitochondria ultrastructure

MCF-7 cells grown on sapphire discs and transfected with *siCtl* or siRNAs specific to *COASY-short isoform* (*siE3-6#1* and *#2*), were high pressure frozen using a Leica HPF (Leica Mycrosystems Vienna, Austria) 36 h after transfection. Freeze substitution was performed in a Leica AFS2 overnight using 2% osmium tetraoxide and 0.1% uranylacetate in acetone with 5% water (49). After three washes with acetone, the samples were infiltrated in Epon resin and polymerized at 60°C for 72 h. Sections of 70 nm thick were cut using a Leica Ultracut UCT microtome (Leica Microsystems) and adhered to formvar coated copper grids. They were stained with 2% uranyl acetate and lead citrate. Then, sections were imaged in a Tecnai T12 electron microscope running at 120 kV and equipped with an Eagle 4k × 4k CCD camera (Thermo Fisher Scientific). These analyses were conducted at the Maastricht Multimodal Molecular Imaging Institute.

### Oxygen consumption rate

All experiments were performed with a Seahorse XFp extracellular flux analyzer (Agilent, Santa Clara, CA, USA). Cells were seeded (8000 cells per well) in XFp mini-plates (Agilent) and allowed to attach overnight. Mitochondrial oxygen consumption rate (OCR) (pmol/min) was measured as previously described (50) on cells kept in un-buffered serum-free DMEM (Basal DMEM, Agilent) supplemented with pyruvate (1 mM), glutamine (2 mM) and glucose (10 mM), pH 7.4 at 37 °C, and ambient CO_2_ for 1 h before the assay. During the assay, cells were successively treated with oligomycin (1 μM), FCCP (1 μM) and rotenone/antimycin A mix (0.5 μM each). OCR was normalized according to cell number evaluated by Hoechst incorporation.

### Cell proliferation

The impact of depleting the COASY-short isoform on cell proliferation was assessed with the IncuCyte® S3 Live-Cell Analysis System (Sartorius, Göttinger, Germany). Cells in 96-well plate were transfected with appropriate siRNAs, after 24 h, the medium was replaced, and cisplatin (50 μM) was added. Triplicates were prepared for each condition. Four or five phase contrast images / well were acquired every 2 h for 48 h with a 10x objective. Measurements were started 30 min after the addition of cisplatin. Cell confluency in each image was assessed by the IncuCyte® software and normalized to the corresponding image taken at *T* = 0. Three independent experiments were run.

### Apoptosis

Apoptosis induced by cisplatin in the different cell lines was assessed by flow cytometry analysis with dual labelling Annexin-V-FITC and Propidium Iodide. The latter two compounds were obtained from Becton and Dickinson (Franklin lakes, NJ, USA) and Sigma respectively. Prior to analysis, MCF-7 cells were detached from growing support with Accutase. Annexin V+/PI– and Annexin V+/PI+ cells were considered apoptotic. A minimum of three independent experiments were performed.

### Protein extraction and western-blot analysis

Cells were lysed in modified RIPA buffer (50 mM Tris–HCl (pH 7.5), 150 mM NaCl, 1 mM EDTA, 1 mM DTT, 1% (v/v) Igepal®CA-630 and 0.25% (w/v) sodium deoxycholate) supplemented with a cocktail of protease inhibitors (Complete™, Roche) and phosphatase inhibitors composed of 1.5 mM NaF, 1 mM Na_3_VO_4_, 25 mM beta-glycerophosphate (Sigma-Aldrich). Protein concentration was assessed by DC™ assay (Bio-Rad, Hercules, CA, USA) and proteins submitted to a denaturing SDS-PAGE and electro-transferred onto a PVDF membrane. After blocking with 5% non-fat dry milk in TBS-Tween, the membranes were incubated with primary antibodies overnight at 4°C, washed in TBS-Tween and incubated in HRP-conjugated secondary antibody. The chemi-luminescence produced by ECL was visualised with a LAS4000 Biomolecular Imager. The signal recorded was quantified by using Image QuantTL software. Each experiment was run a minimum of three independent times. The list of antibodies is provided in Supplementary Table S1.

### Co-immunoprecipitation

Twenty-four h post-transfection with pDEST-Flag–RBM39, MCF-7 cells were treated with cisplatin (50 μM for 24 h) or mock-treated, scraped and lysed on ice for 20 min in IP lysis buffer (20 mM Tris–HCl pH 7.5, 150 mM NaCl, 1% Triton X-100, 10% glycerol) complemented with protease and phosphatase inhibitors. Total extract corresponding to 1 mg of protein was then immunoprecipitated for 2 h at 4°C using control or Flag-antibody in presence of RNAse A followed by incubation with 3 μl of protein A/G magnetic beads (Pierce, Thermo Scientific™) for 1 h at 4°C. After three washes with ice-cold IP lysis buffer, the immunoprecipitated proteins were eluted in 2% SDS, for 10 min at 4°C, denatured at 100°C for 3 min and analysed by western blotting.

### RNA immunoprecipitation followed by RT-qPCR analysis

Twenty-four h after transfection of pDEST-Flag-RBM39, MCF-7 cells were treated or not with cisplatin (50 μM, 24 h). After two washes with ice-cold PBS, the cells were cross-linked with formaldehyde 1% (Sigma) for 10 min at RT. Excess formaldehyde was quenched by glycine 125 mM for 5 min at RT. After washes the cells were collected and lysed in RIPA buffer (50 mM Tris–HCl pH 8.00, 150 mM NaCl, 1.0% Igepal®CA-630, 0.5% sodium deoxycholate, 0.1% SDS) complemented with protease (Complete™), phosphatase (ALT™ phosphatase from Fisher Scientific) and RNAse inhibitor (RNAsin from Promega, Madison, WI, USA), and, sonicated on ice. Small aliquots of equal protein amount were removed and treated with proteinase K RNA grade (Thermo Fisher Scientific) before RNA purification by TRIzol (Thermo Fisher Scientific). These small aliquots were later used to determine RNA inputs in the RT-qPCR. Equal amounts of proteins (∼1 mg of crude extract) were incubated with control or Flag-antibody-coated Dynabeads overnight at 4°C. After washes in RIPA buffer, RNA and proteins were eluted from the magnetic beads by heating 3 min at 90°C in elution buffer (100 mM Tris–HCl pH 8.00, 10 mM Na_2_-EDTA, 1% SDS). Next the proteins were digested by proteinase K RNA grade and RNA was purified with TRIzol. Purified RNAs (from the Inputs and the IPs) were incubated with DNAse 1 then reverse transcribed with Revert AID H minus kit using random primers. Quantitative RT-PCR was conducted in triplicate with a set of selected primers hybridizing in intron 4 and exon 5 of *COASY or in GAPDH* (primers sequences are listed in Supplementary Table S1). RNA enrichments were expressed as % of inputs. Experiments were carried out at least three independent times.

### UV crosslinking RNA-immunoprecipitation followed by RT-qPCR

Twenty-four h after addition of cisplatin (50 μM), MCF-7 cells were washed once with ice-cold PBS and irradiated with UV at 260 nm at 400 mJ/cm^2^ (Stratalinker 1800) on ice. After irradiation, cells were collected, lysed in RIPA buffer (50 mM Tris–HCl pH 8.00, 150 mM NaCl, 1.0% Igepal®CA-630, 0.5% sodium deoxycholate, 0.1% SDS) complemented with protease (Complete™) and phosphatase (ALT™) inhibitors and sonicated (2 × 30 s, 30% power). For each condition, samples (∼1 mg of crude extract) were then treated with RNAse 1 (Invitrogen™ Ambion™, Thermo Fisher) at a final concentration of 5 U/ml for 3 min at 37°C followed by 10 min on ice. RNase inhibitor Ribolock was then added (Thermo Fisher). Input aliquots (10%) were collected and stored at –80°C. Remaining extracts were incubated with control IgG or RBM39 antibody-magnetic coated protein A/G beads overnight at 4°C. After three washes in RIPA buffer, RNA and proteins were eluted from the beads by heating 3 min at 90°C in elution buffer (100 mM Tris–HCl pH8.00, 10 mM Na_2_-EDTA, 1% SDS). The eluted proteins (and the previously stored input aliquots) were digested by proteinase K RNA grade (80 U, 2 × 30 min at 37°C) and finally RNA was purified with TRIzol. Purified RNAs (from the inputs and the IPs) were treated with DNAse 1 and then reversed transcribed and the cDNAs analysed by quantitative PCR as described in the previous paragraph.

### *Gaussia* luciferase proximity complementation assay

Briefly, HEK-293 cells plated in 24-well plates were transfected with 0.5 μg of p-SPICA-N1(N2)-*gaussia* luciferase fused in frame with c-Jun or RBM39 separately or together. After 24 h, cisplatin was added, and the cells grown for an additional 24 h. The cells were then lysed in 200 μl of lysis buffer and the *gaussia* luciferase activity was measured using the *Renilla* luciferase Assay System (Promega), according to manufacturer's instructions.

### Statistics

Means and SD were calculated from at minimum three independent experiments. Statistical significance was determined using two-tailed Student's *t*-test, one-way or two-way ANOVA with GraphPad Prism 8. Significance levels are indicated by *(*P* < 0.05); **(*P* < 0.01); ***(*P* < 0.001); ****(*P* < 0.0001). For RNA seq, adjusted (adj) *P*-value and FDR were calculated using DESeq2 and rMATS respectively. The *P*-value associated to Venn diagram overlaps was determined by the Fisher exact test in the R package GeneOverlap.

## RESULTS

### Cisplatin treatment affects mRNA levels and pre-mRNA splicing

**Figure 1.**
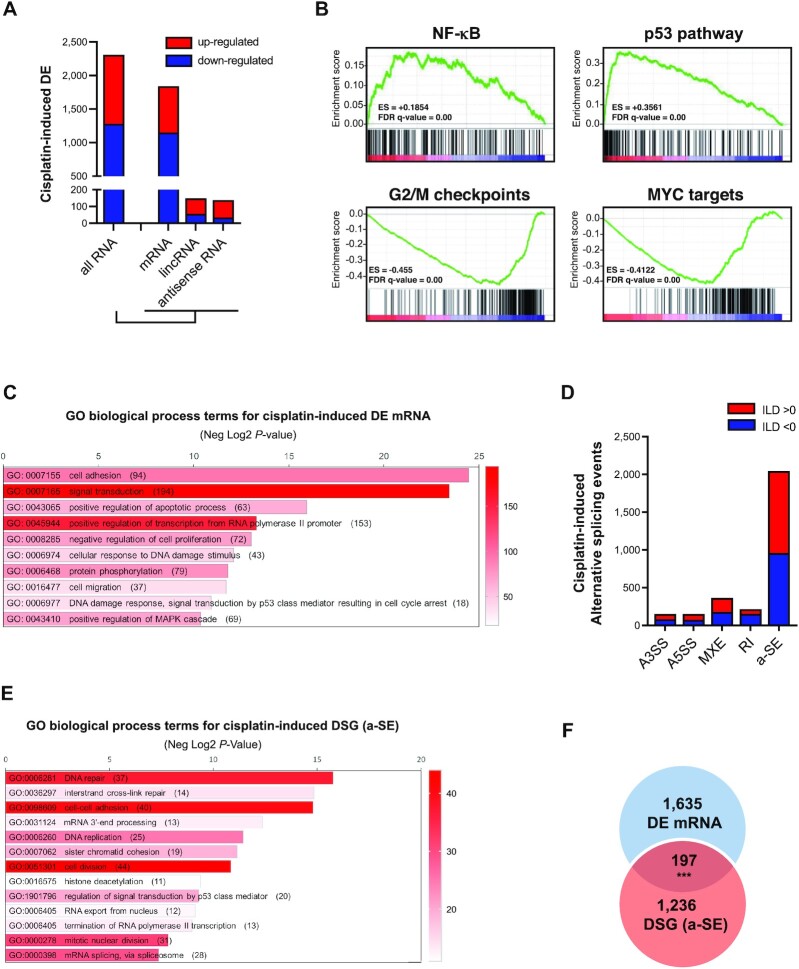
Cisplatin induces large transcriptomic changes in MCF-7 cells, both at the level of gene expression and alternative splicing. (**A**) Number of transcripts up- (red bars, log2(FC) ≥ 1, adjusted *P*-value < 0.05, and TPM ≥ 1 in at least one experimental condition) or down- (blue bars, log2(FC) ≤ 1, adjusted *P*-value < 0.05, and TPM ≥ 1 in at least one experimental condition) regulated after cisplatin treatment. Results are shown for all RNA, protein-coding (mRNA), long intergenic non-coding (lincRNA) and antisense RNA. (**B**) Top GSEA terms associated with differentially expressed mRNA following cisplatin treatment in MCF-7 cells. (**C**) GO Biological Process terms enriched in differentially expressed genes (mRNA) following cisplatin treatment in MCF-7 cells. Number of genes associated with each GO term is indicated in brackets (**D**) Number and type of alternatively spliced (AS) events after cisplatin treatment in MCF-7 cells (FDR < 0.05 and inclusion level difference |ILD| ≥ 0.1, and TPM of ≥ 1 in at least one experimental condition). A3SS: alternative 3’ splice site, A5SS: alternative 5’ splice site, MXE: mutually exclusive exon, RI: retained intron and SE: alternatively spliced exon. Red and blue bars respectively correspond to AS events with positive and negative ILD. A positive ILD is observed when the inclusion level is higher in control than in cisplatin-treated condition. A negative ILD is observed when the inclusion level is lower in control than in cisplatin-treated condition. (**E**) GO Biological Process terms associated with the list of mRNA transcripts with alternative SE following cisplatin treatment in MCF-7 cells. (**F**) Overlap between differentially expressed mRNA (DEG) and differentially spliced mRNA (DSG, *i.e*. transcripts with at least one cisplatin-dependent alternative SE) in MCF-7 cells after treatment with cisplatin. Statistical significance was calculated by Fisher exact test. ***P < 0.001.

To characterize the transcriptomic changes associated with DNA damage in cancer cells we treated MCF-7 breast cancer cells with cisplatin (50 μM, 24 h) and carried out a comparative RNA sequencing (RNA-seq) analysis with untreated controls. MCF-7 cells were chosen because they are considered relatively resistant to this genotoxic agent [(51), https://depmap.org/portal/]. In our experimental conditions, cisplatin treatment led to 23% of cells being apoptotic after 24 h, a percentage that regularly increased with time (Supplementary Figure S1A). For statistical robustness, RNAs from three independent experiments were processed for RNA-seq and investigated for differential gene expression and alternative splicing events. Using stringent filters (*i.e*. |log2 Fold Change (FC)| ≥ 1, adj *P*-value < 0.05 and transcripts per kilobase million (TPM) ≥ 1 in at least one condition), differential analysis of RNA levels between treated and non-treated cells revealed that a total of 2,310 RNAs were differentially expressed (DE) following cisplatin treatment, the large majority of which were mRNAs (1832/2310; 79.3%) (Figure 1A, Supplementary Table S2). Long intergenic non-coding RNA (lincRNA) and antisense RNA accounted for ∼12.2% of the DE RNAs (148/2310; 6.4% and 138/2310; 5.8%, respectively). Globally, slightly more transcripts were downregulated (1280/2310; 55.4%) than upregulated (1030/2310; 44.6%) by cisplatin (Figure 1A). This effect was even more pronounced when looking only at mRNA, with approximately two thirds of transcripts (1149/1832; 62.7%) showing decreased levels after cisplatin treatment. In contrast to coding RNAs, levels of lincRNA and antisense RNAs were generally increased. A Gene Set Enrichment Analysis (GSEA) of DE mRNAs against the ‘Hallmarks MSigDB collection’ identified NF-κB and p53 pathways as the highest positive enrichment scores and MYC targets and G2/M-associated genes as the top negative enrichment scores (Figure 1B). Gene Ontology (GO) analysis revealed that DE mRNAs were enriched in several biological processes compatible with activation of the DDR, including ‘signal transduction’ (GO:0007165), ‘positive regulation of apoptotic process’ (GO:0043065), ‘cellular response to DNA damage stimulus’ (GO:0006974) or ‘DNA damage response, signal transduction by p53 class mediator resulting in cell cycle arrest’ (GO:0006977) (Figure 1C). GO molecular function analysis showed that DE mRNAs were enriched in ‘ATP binding’ (GO:0005524) and ‘protein serine/threonine kinase activity’ (GO: 0004674), all of which were also compatible with the DDR signalling cascade (Supplementary Figure S1B). Cisplatin-dependent changes in steady-state RNA levels were validated by RT-qPCR for a set of 10 RNAs (9 mRNAs and 1 lincRNA), thus validating our DE analytical pipeline (Supplementary Figure S1C).

Alternative splicing analysis uncovered important qualitative changes in the transcriptome of cisplatin-treated cells with a total of 2,922 AS events affecting 1,873 mRNA (Figure 1D and Supplementary Table S2). Alternatively-spliced exons (a-SE, also called cassette exons) were by far the most frequent and accounted for 69.9% (2045/2922) of the total changes and affected 1,433 mRNAs. Among these a-SE, slightly more were included (1086/2045; 53.1%) than excluded (959/2045; 46.9%) upon cisplatin treatment. In most cases (75%, 1074/1433), a single a-SE was found in a given transcript and more rarely two (16%; 236/1433) (Supplementary Figure S1D). Transcripts with more than two splicing events were rare (8.6%; 123/1433). Fifteen cisplatin-dependent a-SE were selected among all significant a-SE presenting an inclusion level difference (ILD) ≥ 0.1 for experimental validation by end-point RT-PCR. In order to detect the amplicons by polyacrylamide gel electrophoresis after a maximum of 30 amplification cycles during PCR, genes with TPM > 20 were selected. Out of these 15 cisplatin-dependent a-SE, 14 were confirmed by RT-PCR (validation rate of 93%, 14/15) after addition of cisplatin (Supplementary Figure S1E and Supplementary Table S1 for primers sequences) thus validating our RNA-seq data and AS analytical pipeline. Messenger RNAs with cisplatin-induced a-SE were significantly enriched in biological processes associated with DNA repair (GO:0006281) and replication (GO:0006260), inter-strand cross-link repair (GO:0036297), cell-cell adhesion (GO:0098609) and mRNA processing, including splicing (GO:0000398) (Figure 1E). Remarkably, only 10.7% (197/1832) of the genes whose expression was affected by cisplatin contained a cisplatin-dependent a-SE (Figure 1F). In addition, GO terms associated with differentially-spliced genes (DSG) with a-SE were different than those associated with DEG, indicating that transcriptional and post-transcriptional (*i.e*. splicing) effects of cisplatin target distinct sets of genes (compare Figure 1C with Figure 1E).

### Cisplatin induces expression of a short isoform of COASY lacking E4 and 5

**Figure 2.**
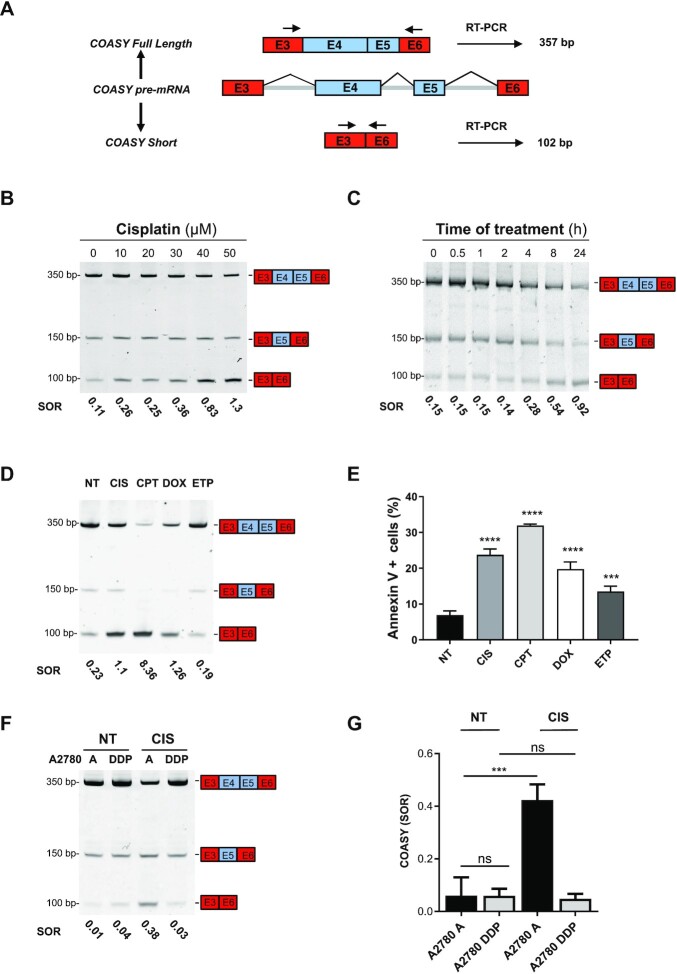
COASY-short isoform lacking E4 and E5 is induced by cisplatin and other DNA damaging agents. (**A**) Schematic representation of the region of the COASY transcript from E3 to E6 with the splice variants containing (COASY-FL) or lacking (COASY-short) E4 and E5. E4 and E5 coordinate in human genome hg38 are chr17:42564729–42564898 and 4256493–4256047 respectively. Constitutive exons are in red and cassette exons are in blue. The arrows indicate the position of the primers used in end-point PCR leading to the amplification of either a 357 bp fragment when E4–5 are included or a 102 bp fragment when E4–5 are skipped in COASY-FL and COASY-short respectively. (**B**) Detection of FL and short *COASY* isoforms in MCF-7 cells treated with the indicated concentrations of cisplatin for 24 h. RNA was extracted, reverse-transcribed and amplified by end-point PCR with primers shown in (A). Amplification products were discriminated by gel electrophoresis. The number below the gel indicate the spliced-out ratio (SOR): short/FL. SOR quantifications based on three independent experiments are shown in Supplementary Figure S2C. (**C**) Detection of FL and short *COASY* isoforms in MCF-7 cells treated with cisplatin (50 μM) for the indicated times. Analysis was performed as described in (B). SOR quantifications based on three independent experiments are shown in Supplementary Figure S2D. (**D**) Detection of FL and short *COASY* isoforms in MCF-7 cells untreated (NT) or treated with cisplatin (CIS, 50 μM), camptothecin (CPT, 5 μM), doxorubicin (DOX, 2 μM) or etoposide (ETP, 50 μM) for 24 h. Analysis was performed as described in (B). (**E**) Apoptosis was detected by flow cytometry analysis of MCF-7 cells treated as described in (D). Percentages of Annexin V^+^ cells are indicated. Histograms represent means of Annexin V + cells ± SD measured from three independent experiments. Statistical significance was calculated by unpaired Student's *t-*test (*n* = 3). ***P< 0.001, ****P< 0.0001. (**F**) *COASY* E4–5 exclusion was assessed in two isogenic ovarian cell lines sensitive (A2780 A) or resistant (A2780 DDP) to cisplatin before (NT) or after (CIS) treatment with cisplatin (50 μM, 24 h). PCR analysis was performed as described in (B). Picture illustrates one representative experiment out of three. (**G**) SOR quantifications from three independent experiments as described in (F). Histograms represent means ± SD. Statistical significance was calculated by Two-Way ANOVA with Bonferroni's multiple comparison test (ns: not significant, ***P< 0.001).

While trying to link the effects of cisplatin on AS to mechanisms of resistance, we turned our attention to genes linked to mitochondrial functions. Indeed, treatment with cisplatin induces mitochondrial dysfunction and several lines of evidence support a prominent role for mitochondria in cisplatin resistance, although the exact mechanism remains incompletely defined (22,24,52,53). Interestingly, we found that almost 10% (132/1433, 9.2%) of transcripts undergoing cisplatin-mediated a-SE code for proteins listed in MitoCarta 3.0, a database of human mitochondrial proteins and pathways. By contrast, only 4.5% of DEG (84/1832) encode proteins that are present in the MitoCarta database, suggesting that cisplatin might preferentially affect mitochondria-related transcripts via AS. Among the 132 alternatively spliced transcripts that were related to mitochondria function, we focused on *COASY*, which codes for Coenzyme A synthase (36). COASY is important for proliferation in various cancer cell lines (www.depmap.org) and plays a role in the sensitivity of rectal cancer cells to radiation-induced DNA damage (40,41). Our RNA-seq analysis revealed that upon treatment with cisplatin, *COASY* undergoes a complex splicing event involving skipping of two consecutive cassette exons (exons 4 and 5, E4–5) (Supplementary Figure S2A) while its expression level remains unchanged both at mRNA (green dot in Supplementary Figure S1C) and protein levels (Supplementary Figure S2B). Skipping of E4–5 in the *COASY* transcript generates a shorter COASY isoform, in frame with the full-length isoform, but lacking the unique ATP binding site and thus, devoid of catalytic activity. First, we validated skipping of *COASY* E4–5 using an isoform-discriminating end-point RT-PCR with primers located in upstream (exon 3) and downstream (exon 6) exons (Figure 2A). This approach allowed us to simultaneously amplify *COASY* mRNA isoforms lacking and containing exons 4 and 5 (hereafter referred as COASY-short and COASY full length (FL), respectively) and to calculate a spliced-out ratio (SOR, *i.e*. short/FL). While MCF-7 cells predominantly express the FL isoform, as indicated by SOR values (<1; usually below 0.15), treatment with cisplatin led to a dose- and time-dependent increase in the proportion of the short variant (Figure 2B, C and Supplementary Figure S2C,D). An effect of cisplatin on inclusion of E4–5 was already detectable at 20 μM and as early as 4 h after starting the treatment. The skipping of E4 alone is not affected by increasing concentration of cisplatin and leads to a frame shift. Cisplatin-induced skipping of COASY E4–5 was not restricted to breast cancer cells, as it was also observed in several other cancer cell lines as well as primary endothelial cells (HUVECs, human umbilical endothelial cells) (Supplementary Figure S2E). Interestingly, the strongest effects on COASY E4–5 inclusion, leading to large accumulation of the short isoform (*e.g*. SOR values > 1) were associated with elevated apoptosis (Supplementary Figure S2F). In addition, skipping of COASY E4–5 was also induced by other DNA-damaging agents, albeit to different extents (Figure 2D). H_2_O_2_-induced oxidative stress had no effect on COASY E4–5 splicing (Supplementary Figure S2G). Again, the intensity in the change of the COASY E4–5 splicing pattern was correlated with the effect of the DNA-damaging agent on cell viability. The strongest effects on COASY E4–5 inclusion were observed for camptothecin (5 μM, SOR = 5.28) and doxorubicin (2 μM, SOR = 1.32), which also exhibited the highest toxicities towards MCF-7 cells (Figure 2E). In contrast, etoposide (50 μM) or H_2_O_2_ (500 μM) had lower impact on MCF-7 viability and no effect on E4–5 inclusion levels (Figure 2E and Supplementary Figure S2H). These observations suggested that the splicing switch between the long (*i.e*. E4–5 containing) and the short (*i.e*. lacking E4–5) isoforms might be involved in sensitivity towards DNA-damaging agents. To test this, we compared the splicing pattern of *COASY* E4–5 in isogenic pairs of cisplatin-sensitive and resistant ovarian carcinoma (A2780 A and A2780 DDP, respectively) and ileocecal adenocarcinoma (HCT8 A and HCT8 DDP, respectively) cell lines. In both cell types, skipping of COASY E4–5 was increased in the sensitive cells in response to cisplatin, leading to more *COASY*-short isoform (Figure 2F, G and Supplementary Figure S2I, J). In contrast, *COASY* E4–5 splicing remained unaffected by cisplatin in the resistant cells with SOR of 0.048 and 0.061 for A2780 and HCT8, respectively. Altogether, these observations indicate that expression of the short isoform of *COASY* correlates with cisplatin-induced cell death.

### Downregulation of COASY short isoform lacking E4–5 alters mitochondria shape, function and sensitivity to cisplatin

**Figure 3.**
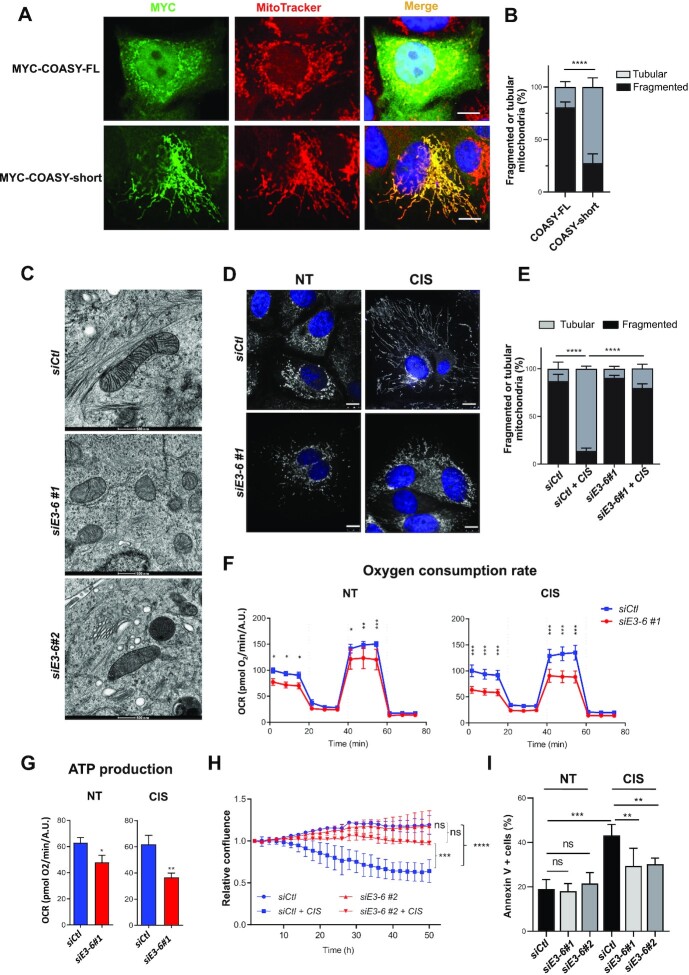
COASY-short isoform induces mitochondrial fusion and apoptosis in MCF-7 cells. (**A**) Immunofluorescence of MYC-COASY-FL or MYC-COASY-short (in green) and mitochondria staining by MitoTracker (in red) in MCF-7 cells transiently transfected. Nuclei are stained in blue by Hoechst 33342. Scale bar: 10 μm. (**B**) Quantification of the percentage of cells with tubular or fragmented mitochondrial network (n > 300) as observed in (A). Histograms are means ± SD. Statistical significance was calculated with Two-Way ANOVA with Bonferroni's multiple comparison test (****P< 0.0001). (**C**) Representative cryo-EM images of mitochondrial ultrastructure in MCF-7 cells transfected with control siRNA (*siCtl*) or two alternative siRNA against *COASY*-short isoform targeting the junction of exon 3 and exon 6 (*siE3-6#1* or #*2*) (magnification 11 000×, scale bar: 500nm). (**D**) Immunofluorescence of mitochondria stained by MitoTracker in MCF-7 cells transfected with a control siRNA (*siCtl*) or a siRNA specific for *COASY-short* (*siE3-6#1*) and subsequently treated (CIS) or not (NT) with cisplatin (50 μM, 24 h). (**E**) Quantification of the percentage of cells with tubular or fragmented mitochondrial network from images (n > 300) as described in (D). Statistical significance was calculated with Two-Way ANOVA with Bonferroni's multiple comparison test (****P< 0.0001). (**F**) Oxygen Consumption Rate (OCR) and (**G**) ATP production-related OCR in cells transfected with control (*siCtl*) or COASY-short siRNA (*siE3-6#1*) and untreated (NT) or treated (CIS) with cisplatin (50 μM, 24 h). OCR were normalised according to Hoechst incorporation (A.U.). Statistical significance of kinetic OCR was calculated with Two-Way ANOVA followed by Bonferroni's multiple comparison (*P< 0.05, **P< 0.01, ***P< 0.001). Statistical analysis of ATP production-related OCR was performed by Student's *t*-test. (**H**) Growth rates of cells transfected with control (*siCtl*) or COASY-short siRNA (*siE3-6#2*) and untreated or treated (+ CIS) with cisplatin (50 μM) were monitored by confluence analysis using live imaging (IncuCyte) over 48h. Results are means ± SD of confluence values normalised to the confluence at T = 0, from three independent experiments run in three technical replicates for each condition. Statistical significance was calculated with Two-Way ANOVA with Bonferroni's multiple comparison test (ns: not significant, ns > 0.05, ***P< 0.001, ****P< 0.0001). (**I**) Apoptosis was measured by flow cytometry using the Annexin V/Propidium Iodide method in cells transfected with control (*siCtl*) or COASY-short isoform siRNAs (*siE3-6#1* or *siE3-6#2*) and untreated (NT) or treated (CIS) with cisplatin (50 μM, 24 h). Percentages of Annexin V^+^ cells indicative of early and late apoptosis are indicated. Histograms are means ± SD from three independent experiments. Statistical significance was calculated with Two-Way ANOVA with Bonferroni's multiple comparison test (ns: not significant, **P< 0.01, ***P< 0.001).

To determine whether the COASY-short isoform plays a role in the sensitivity to cisplatin, we modulated its levels by ectopic expression or knockdown experiments. Immunofluorescence imaging revealed that ectopically-expressed COASY-FL or -short isoform mainly localised in mitochondria (Figure 3A) (37). However, these experiments also revealed that overexpression of COASY-short dramatically modified the mitochondrial network that appeared as elongated interconnected strings. Qualitative visual inspection and counting of cells with a ‘tubular’ or ‘fragmented’ phenotype, an approach routinely used to evaluate mitochondrial morphology (54) indeed confirmed that expression of the short isoform tilted the fission/fusion balance in favour of fusion and a ‘tubular’ morphology (Figure 3B). Based on these observations, we next sought to assess the role of *COASY*-short isoform in cisplatin-induced changes in mitochondrial dynamics and function by a loss-of-function approach. To this aim, we designed two siRNA against the E3-E6 junction, thus allowing specific targeting of the short isoform with little or no effect on the full-length transcript (Supplementary Figure S3A) and protein (Supplementary Figure S3B) for siRNAs *E3-6#1* and *#2* respectively. Inspection of mitochondrial ultrastructure by cryo-electron microscopy uncovered important morphological alterations in cells knocked down for *COASY-*short, which showed a decrease in cristae density and mitochondria swelling for both siRNAs (Figure 3C). As reported in other cancer cell types, cisplatin promoted mitochondrial fusion and dramatically affected the mitochondrial network of control MCF-7 cells, with the percentage of cells exhibiting tubular mitochondria going from approximately 20% in the absence of cisplatin to more than 80% following exposure to cisplatin (Figure 3D, E and Supplementary Figure S3C) (55). Remarkably, the effects of cisplatin on the fission/fusion dynamics were abolished in cells where expression of *COASY* lacking E4–5 was prevented by a specific siRNA, as most of these cells retained a fragmented mitochondrial network upon treatment with cisplatin. Fusion is crucial in the maintenance of high mitochondrial activity (56). Notably, ectopic expression of MYC-COASY-FL in cisplatin-treated cells was unable to prevent mitochondrial changes induced by the treatment (Supplementary Figure S3D and S3E for quantification). Altogether, these experiments suggest that cisplatin-induced mitochondrial changes are most likely due to the expression of COASY-short, rather than to a reduction in the levels of COASY-FL. Supporting this, COASY-FL abundance is not significantly affected by cisplatin (see Supplementary Figure S2B). Because the COASY-short isoform promotes mitochondrial fusion, we next assessed mitochondrial function in cells depleted for COASY lacking E4–5 by measuring oxidative phosphorylation. Compared to control cells, kinetic normalized oxygen consumption rate (OCR), including ATP production-related OCR were reduced in the absence of the COASY-short isoform, both in untreated or cisplatin-treated cells (Figure 3F, G). Altogether, these observations demonstrate that depletion of the short isoform of COASY decreases mitochondrial fusion and activity. Several independent studies have shown that high mitochondrial metabolic activity significantly contributes to cisplatin cytotoxicity (22,57). Based on our findings, we thus postulated that depletion of COASY-short isoform would render cells less sensitive towards cisplatin. While depletion of COASY-short isoform had no effect on MCF-7 health and viability in the absence of cisplatin, it significantly reduced the cytotoxic effects of cisplatin and increased cell resistance (Figure 3H, I). As illustrated for COASY, our data highlight a model in which changes in AS events induced by cisplatin generate mRNA isoforms (*e.g*. COASY-short) that contribute to its toxicity.

### Inactivation of RBM39 mediates a large proportion of the effects of cisplatin on AS.

**Figure 4.**
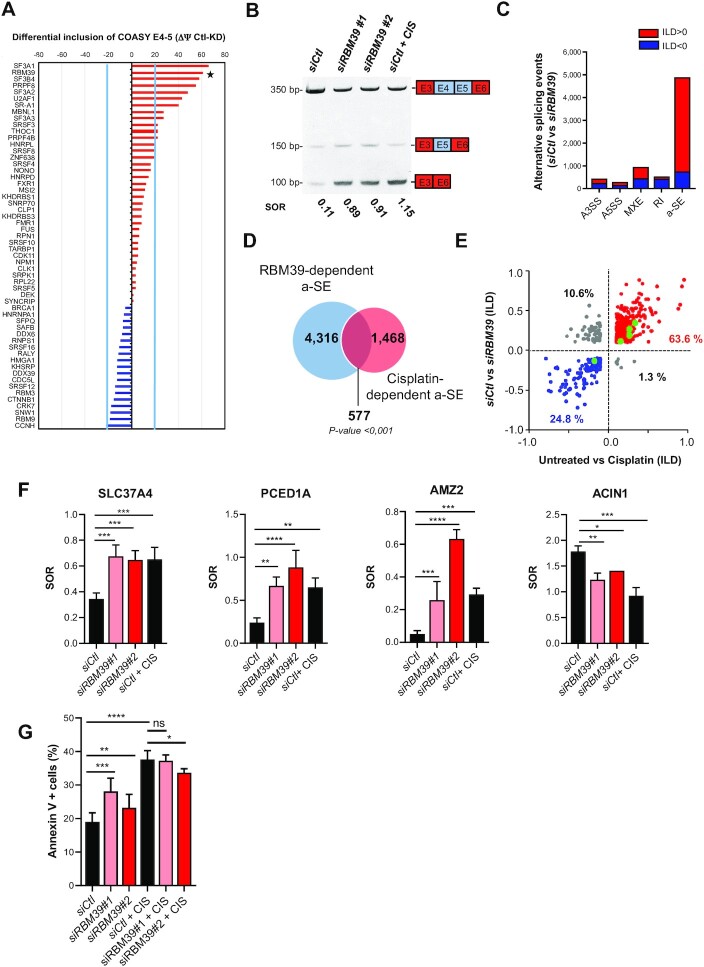
A large portion of cisplatin-dependent modified SE are regulated by RBM39. (**A**) MCF-7 cells were transfected with siRNAs against the indicated splicing factors (SFs) or a control siRNA. After 72 h, inclusion of E4–5 of *COASY* was evaluated by end-point RT-PCR. The abundance of the short variant lacking exon 4 and exon 5 was expressed as the difference in percent spliced-in between the control and the SF-knocked down cells (ΔΨ = PSI_Ctl_– PSI_KD_). ΔΨ values > 0 (in red), indicate that knocked-down (KD) of the SF favours the exclusion of E4–5. ΔΨ values < 0 (in blue) indicate that KD of the SF favours the inclusion of E4–5. Arbitrary ΔΨ cut-offs of +20% and –20% are indicated by light blue lines. A black star indicates the position of RBM39. (**B**) Detection of FL and short *COASY* isoforms in MCF-7 cells transfected with control siRNA (si*Ctl*) or RBM39 siRNA *(siRBM39#1* or *siRBM39#2*) and treated (CIS) or not with cisplatin (50 μM, 24 h). RNA was extracted, reverse-transcribed and amplified by end-point PCR. Amplification products were discriminated by gel electrophoresis. The numbers below the gel indicate the spliced-out ratio (SOR): short/ FL. Picture illustrates one representative experiment. SOR quantifications based on three independent experiments are shown in Supplementary Figure S4B. (**C**) Numbers and types of AS events following RBM39 knockdown in MCF-7 cells (FDR < 0.05 and |ILD| ≥ 10% and TPM ≥ 1 in one experimental condition. A3SS: alternative 3’ splice site, A5SS: alternative 5’ splice site, MXE: mutually exclusive exon, RI: retained intron and SE: alternatively spliced exon. Red and blue bars correspond to AS events with respectively positive and negative ILD (ILD ≥ 10%) corresponding to a decreased or an increased inclusion in the *siRBM39-*treated condition compared to the *siCtl* condition. (**D**) Venn diagram showing the overlap between RBM39-dependent a-SE and cisplatin-dependent a-SE in MCF-7 cells with a *P*< 0.001 determined by Fisher exact test. (**E**) Correlation between the ILD in response to cisplatin (X-axis) or in response to RBM39 knockdown (Y-axis) of the 577 common a-SE shown in (D). The red and blue dots have respectively a positive and negative ILD in both conditions. Grey dots illustrate a-SE with inverse regulation by cisplatin or RBM39 knockdown. Larger green dots are COASY and four random a-SE selected for cross-validation by RT-PCR in (F). (**F**) a-SE in *SLC37A4* (exclusion of E7) (n = 4), *PCED1A* (exclusion of E7) (n = 4), *AMZ2* (exclusion of E3) (n = 3) and ACIN1 (exclusion of E4) (n = 3) were selected for validation by RT-PCR. Histograms are means ± SD of SOR from three or four independent experiments as indicated. Statistical significance was calculated by One-Way ANOVA (*P< 0.05, **P< 0.01, ***P< 0.001, ****P < 0.0001). (**G**) Apoptosis was measured by flow cytometry using the Annexin V/Propidium Iodide method in cells transfected with control (*siCtl*) or RBM39 siRNA (*siRBM39#1* or *siRBM39#2*) and untreated or treated (+CIS) with cisplatin (50 μM, 24 h). Percentage of Annexin V^+^ cells was measured. Histograms are means ± SD from four independent experiments. Statistical significance was calculated with Two-Way ANOVA with Bonferroni's multiple comparison test (ns: not significant, * *P*< 0.05, ** *P*< 0.01, ***P< 0.001, ****P< 0.0001).

Next, we used the skipping of E4–5 of *COASY* as a prototypical event to unravel the molecular mechanisms of AS regulation by cisplatin. Only 9 out of the 1,832 DE mRNA (3 up- and 6 down-regulated) by cisplatin were annotated as ‘mRNA splicing via spliceosome’ (GO:0000398). This made it very unlikely that cisplatin indirectly impacted AS by modulating expression of splicing regulators, although it remained a possibility. To identify potential effectors of cisplatin-driven AS, we knocked down 56 potential splicing regulators using previously validated siRNA [(58) and Supplementary Table S1 for sequences] and monitored the inclusion of E4–5 of COASY by RT-PCR in non-treated MCF-7. Differences in COASY E4–5 inclusion were calculated as the difference in percent spliced-in [PSI (Ψ) = FL/(FL + short) × 100] between knocked down (KD; *i.e*. siRNA-treated) and control (*siCtl*-treated) cells conditions (ΔΨ = PSI_Ctl_ – PSI_KD_) (Figure 4A and Supplementary Table S3). To maximize our chances to identify relevant splicing factors, we only considered knockdowns that induced splicing change above 20% E4–5 PSI (|ΔΨ| > 20%). Strikingly, these were all knockdowns that decreased E4–5 inclusion, similarly to cisplatin treatment. Among these, the most pronounced effects were seen for SF3A1, RBM39 and SF3B4. SF3A1 and SF3B4 are both U2 snRNP-associated splicing factors and depleting these general splicing factors would be expected to non-specifically affect the splicing of many alternative exons. RBM39 is a well-known RNA-binding protein that has been shown to regulate alternative splicing of several genes (10,15,18). We thus decided to focus our attention on RBM39 and test the hypothesis that it might be involved in cisplatin-dependent regulation of specific AS.

First, we verified that depletion of RBM39 promoted exclusion of E4–5 similarly to cisplatin, using two different siRNA (Figure 4B, Supplementary Figure S4A,B). To test whether this effect was restricted to COASY, we knocked down RBM39 with *siRBM39#2* and profiled associated splicing changes by RNA-seq analysis on a genome-wide scale (*n* = 3). Downregulation of RBM39 had a large impact on AS, as it led to a total of 7,131 AS events (Figure 4C, Supplementary Figure S4C and Supplementary Table S4). Among these, the most frequent were by far cassette exons [SE, 68.6% (4893/7131)] with a large dominance of exclusion (84.4% (4134/4893). The quality of our RNA-seq analytical pipeline was experimentally validated by end-point RT-PCR on 15 a-SE events (including COASY) (Supplementary Figure S4D). Thirteen out of these 15 events (86%) were cross-validated by RT-PCR. Thirteen a-SE events were also confirmed with the second siRBM39 (Supplementary Figure S4E). By comparing RBM39-dependent and cisplatin-induced SE, we identified 577 common events on 468 independent genes, which accounted for 28.2% (577/2045) of all cisplatin-induced SE, indicating that almost one third of cisplatin-induced a-SE are also controlled by RBM39 (Figure 4D). Strikingly, 88% (24.8% with reduced exclusion and 63.6% with increased exclusion) of these 577 common events (507/577) were similarly affected by RBM39 knockdown or cisplatin treatment, while only 12% were oppositely regulated (*i.e*. more excluded in response to cisplatin but more included in response to *siRBM39*) (Figure 4E). Examples of similar positive and negative qualitative transcriptional regulation by RBM39 depletion and/or cisplatin treatment were validated by RT-PCR analysis for a series of SEs (Figure 4F).

In addition to AS, the depletion of RBM39 modified the expression of 836 mRNAs. In agreement with the literature describing RBM39 as a positive regulator of ER and AP-1, GSEA analysis indicated that RBM39 depletion downregulated oestrogen and AP-1 responses (Supplementary Figure S4F). Negative enrichment scores for G2/M and MYC targets were also seen after RBM39 depletion. Overall, there was a significant overlap of 252 genes between the DE mRNA following RBM39 knockdown and the DE mRNA after cisplatin treatment (Supplementary Figure S4G). This represents 13% (252/1832) of all DE mRNA induced by cisplatin. The vast majority [88%, (155 + 66)/252] of these common DE mRNA were modified in the same direction by cisplatin and RBM39 depletion (26.2% were upregulated and 61.5% downregulated) (Supplementary Figure S4H).

Altogether, these observations show that depletion of RBM39 recapitulates many of the transcriptomic changes seen upon treatment with cisplatin, both at the gene expression and mRNA splicing levels.

Based on our observations that (i) depletion of RBM39 recapitulates a significant proportion of cisplatin-dependent a-SE and (ii) induction of specific splicing isoforms, including COASY-short by cisplatin plays a role in its cell toxicity, we assessed the effect of depleting RBM39 on MCF-7 survival. Similarly to cisplatin, knocking down RBM39 induced apoptosis in MCF-7 cells. Moreover, the mortality induced by cisplatin was not further enhanced by RBM39 downregulation (Figure 4G).

### c-Jun interacts with RBM39 and prevents its binding to the COASY pre-mRNA in response to cisplatin

Because depletion of RBM39 and cisplatin treatment had a similar effect on inclusion of COASY E4–5, we postulated that cisplatin might lead to RBM39 inactivation. To test this hypothesis, we first assessed the effect of cisplatin on RBM39 mRNA and protein levels. Western blot analysis showed that protein levels of RBM39 were unaffected by cisplatin (Supplementary Figure S5A, B). At the mRNA level, cisplatin did not reduce *RBM39* mRNA steady-state levels, thus ruling out the trivial explanation that cisplatin might inhibit RBM39 splicing function by reducing its cellular levels (Supplementary Figure S5C). Immunofluorescence analysis also revealed that cisplatin did not notably alter RBM39 intracellular localisation (Supplementary Figure S5D).

**Figure 5.**
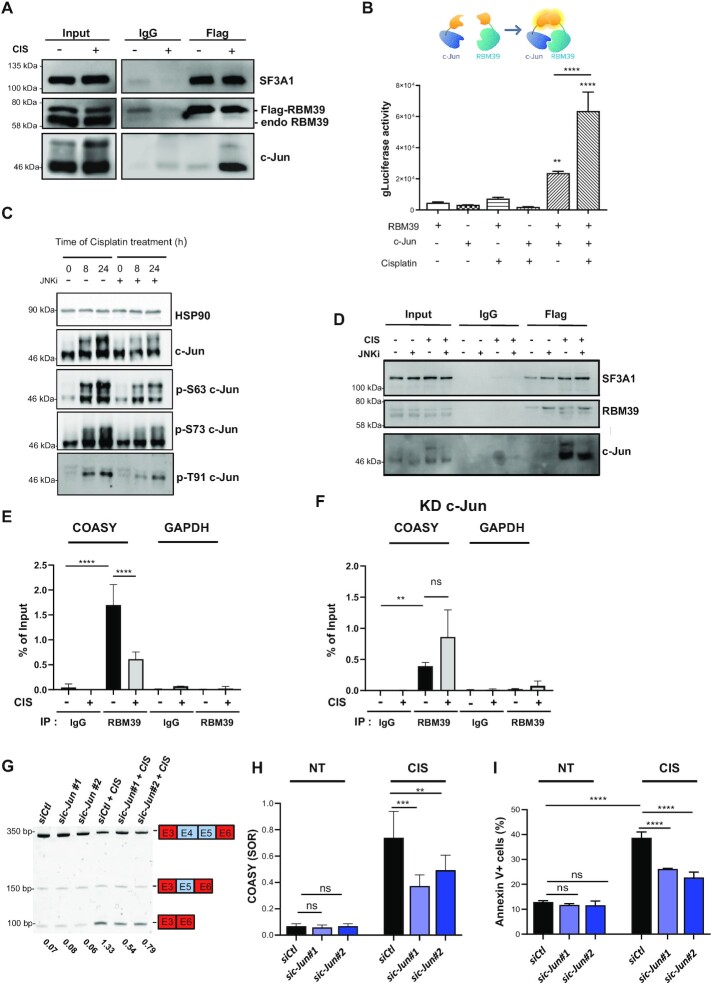
Association between c-Jun and RBM39 is promoted by cisplatin and controls *COASY* E4–5 exclusion by reducing RBM39 binding to the *COASY* pre-mRNA. (**A**) Cells overexpressing Flag-RBM39 were treated (+) or not (–) with cisplatin (CIS). Total protein extracts were immunoprecipitated with control IgG or anti-Flag-antibodies in presence of RNAse. Co-immunoprecipitation of c-Jun and SF3A1 with Flag-RBM39 was assessed by western blotting using the indicated antibodies. (**B**) Protein interaction assay between RBM39 and c-Jun, using the *gaussia* princeps luciferase complementation method. Results are means ± SD from three independent experiments. (**C**) Western blotting of c-Jun using phospho-specific antibodies in MCF-7 cells treated with cisplatin (50 μM) for the indicated times and pre-treated (+) or not (–) with the JNK inhibitor (JNK-IN-8, 1 μM), 3 h prior to cisplatin treatment. HSP90 was used as loading controls. (**D**) Cells overexpressing Flag-RBM39 were treated (+) or not (–) with cisplatin (CIS, 50 μM, 24 h) and pre-treated or not with the JNK inhibitor, 3 h before adding the cisplatin. Total protein extracts were immunoprecipitated with control IgG or anti-Flag-antibodies in presence of RNAse. Co-immunoprecipitation of c-Jun and SF3A1 with Flag-RBM39 was assessed by western blotting using the indicated antibodies. (**E**) MCF-7 cells were treated (+) or not (–) with cisplatin (CIS, 50 μM, 24 h). The presence of RBM39 on the *COASY* pre-mRNA was assessed by UV-cross-linked RNA immunoprecipitation using either control IgG or anti-RBM39 antibodies. *GAPDH* was used as a negative control. Results are expressed as means ± SD from three independent experiments, expressed relative to levels of the RNAs in inputs. Statistical significance was calculated by One-Way ANOVA (****P< 0.0001). (**F**) Same as in (E) but MCF-7 cells were depleted from c-Jun by siRNA 24 h prior to the addition of cisplatin. (**G**) Detection of FL and *COASY* lacking E4–5 isoforms in MCF-7 cells transfected with control (*siCtl*) or one of two independent siRNA against c-Jun (*sic-Jun#1* or *sic-Jun#2*) and treated (CIS) or not (NT) with cisplatin (50 μM, 24 h). A representative acrylamide gel electrophoresis is shown. (**H**) Quantifications of SOR (short/FL) from three independent experiments as shown in (G). Histograms are means SOR ± SD. Statistical significance was calculated by Two-Way ANOVA (ns: not significant, **P< 0.01, ***P< 0.001, ****P < 0.0001). (**I**) Apoptosis was measured by flow cytometry using the Annexin V/Propidium Iodide method in cells transfected with control (*siCtl*) or one of two independent c-Jun siRNA (*sic-Jun#1* or *sic-Jun#2*) and untreated (NT) or treated (CIS) with cisplatin (50 μM, 24 h). Percentage of Annexin V + cells was measured. Histograms are means ± SD from three independent experiments. Statistical significance was calculated with Two-Way ANOVA with Bonferroni's multiple comparison test (ns: not significant, ****P< 0.0001).

While investigating the connections between cisplatin and RBM39, we turned our attention towards c-Jun, a major component of the dimeric AP-1 TF. Indeed RBM39 binds specifically to c-Jun and acts as a transcriptional co-activator for AP-1 (16). In addition, c-Jun is activated by cisplatin via phosphorylation of its trans-activation domain (TAD) and transcriptional up-regulation and participates in mechanisms of cisplatin resistance in several types of cancer (59–62). Together with the recent finding that some TFs partner with RBPs to control splicing events, these observations led us to hypothesize that c-Jun might interfere with RBM39-dependent splicing (7,8). First, we monitored the interaction between c-Jun and RBM39 in untreated and cisplatin-treated MCF-7 cells using co-immunoprecipitation experiments. No co-immunoprecipitation was detected between c-Jun and RBM39 in the absence of cisplatin in our experimental conditions (Figure 5A). However, treatment with cisplatin led to a robust association between both proteins. In contrast, the association between RBM39 and SF3A1, a U2 snRNP component was detected in untreated cells and was not affected by cisplatin. We noticed that c-Jun protein levels were slightly increased by cisplatin treatment. A small up-regulation of c-Jun by cisplatin was also observed at the level of mRNA (Supplementary Figure S5E). To verify that the cisplatin-induced association between RBM39 and c-Jun was not a mere reflection of the increased levels of c-Jun, we validated these results with an alternative protein-protein interaction assay based on a proximity complementation of the *gaussia* luciferase enzymatic activity (gPCA) (Figure 5B). Although the levels of recombinant N1-gLuc-RBM39 and N2-gLuc-c-Jun remained largely unaffected by the presence of cisplatin (Supplementary Figure S5F), the association between RBM39 and c-Jun was again promoted by cisplatin.

As a TF, c-Jun is activated by phosphorylation of its N-terminal TAD domain at residues S63/S73 and T91/T93 by the c-Jun N-terminal kinase (JNK), which is thought to promote its interactions with co-activators (63). In agreement with previous reports, immunoblotting analysis using phospho-specific antibodies for S63, S73 and T91 showed that cisplatin induces a persistent phosphorylation of c-Jun at these residues in MCF-7 cells (Figure 5C) (62). However, adding the JNK inhibitor JNK-IN-8 reduced cisplatin-induced phosphorylation of c-Jun but did not affect its ability to interact with RBM39 in response to cisplatin (Figure 5D).

Using RNA immunoprecipitation experiments with reversible formaldehyde cross-linking between RNA and protein (RIP), we verified that Flag-RBM39 associates with the *COASY* pre-mRNA in untreated MCF-7 cells (Supplementary Figure S5H). Quantitative RT-PCRs were run with primers selected in intron 4 and exon 5 of COASY. However, association of RBM39 with the *COASY* pre-mRNA was significantly reduced following cisplatin treatment. Next, we performed similar RIP experiments in c-Jun depleted cells (Supplementary Figure S5I). Although for unknown reasons the basal association (*i.e*. in the absence of cisplatin) of RBM39 with the *COASY* pre-mRNA was slightly reduced in c-Jun KD cells, this association was largely insensitive to cisplatin treatment, supporting the idea that c-Jun is responsible for the cisplatin-induced detachment of RBM39 from the *COASY* pre-mRNA Because formaldehyde fixation can lead to the detection of indirect RNA-protein interactions, we next performed RNA-immunoprecipitation with UV-crosslinking and partial RNA digestion (CLIP). In this approach, only RNAs directly bound by the RBP are captured. In agreement with the previous RIP experiments, we observed the recruitment of endogenous RBM39 to *COASY* pre-mRNA and a decreased recruitment in presence of cisplatin in cells expressing c-Jun (Figure 5E). This decrease in RBM39 binding to COASY was not detected in c-Jun KD cells (Figure 5F).

To confirm this model, we assessed the impact of depleting c-Jun on the splicing of E4–5 of *COASY*. Knocking down c-Jun in the absence of cisplatin had no impact on E4–5 inclusion (Figure 5G,H and Supplementary Figure S5G). However, downregulation of c-Jun by *sic-Jun* significantly reduced the proportion of the short variant in response to cisplatin, establishing the importance of c-Jun in cisplatin-mediated AS of COASY. In agreement with these observations, we found that depletion of c-Jun had no impact on cell survival in basal cell death but significantly reduced the sensitivity of MCF-7 towards cisplatin (Figure 5I). Altogether, our results are consistent with the hypothesis that interaction with c-Jun, which is induced by cisplatin interferes with RBM39 splicing activity and promotes skipping of E4–5 from the *COASY* mRNA.

### c-Jun participates in genome-wide cisplatin-induced AS, independently of its transcriptional activity

**Figure 6.**
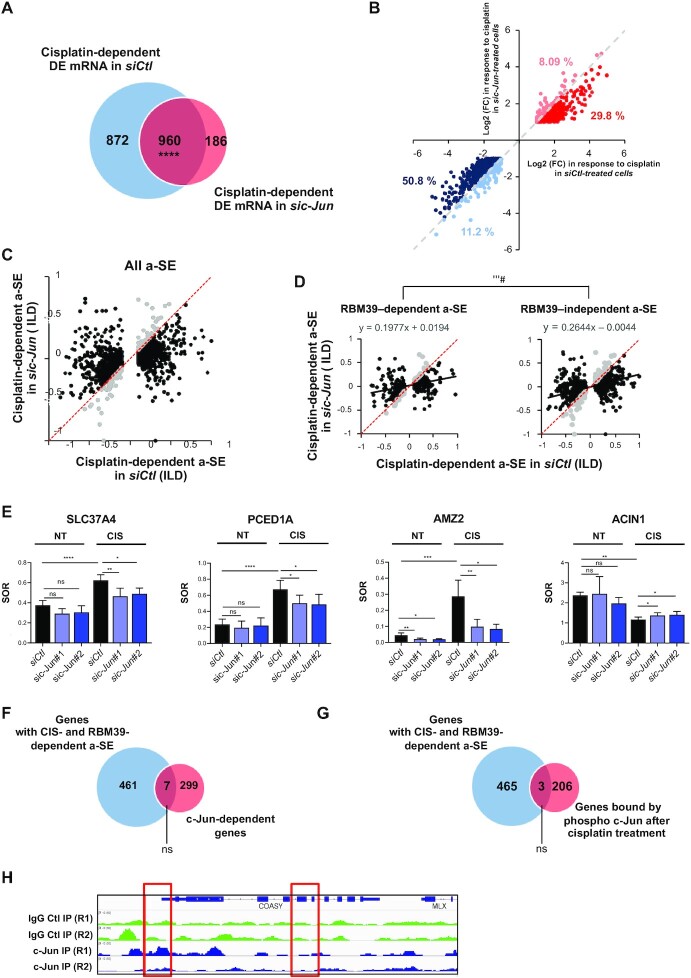
c-Jun participates in the effects of cisplatin on gene expression and alternative splicing. (**A**) Venn Diagram representing the overlap between cisplatin-dependent DEG in MCF-7 cells treated with a control siRNA (*siCtl*) or a siRNA against c-Jun (*sic-Jun*). Only genes (mRNA) with a log2(FC)≥1 or ≤-1, adjusted *P*-value < 0.05 and TPM ≥ 1 in at least one condition (NT or CIS) in both control and depleted cell are considered. (**B**) Correlation between the log2(FC) in response to cisplatin in *siCtl*-treated (X-axis) or *sic-Jun*-treated (Y-axis) MCF-7 cells of the 960 common DEG shown in (A). The red and blue dots have respectively a positive and negative log2(FC) in response to cisplatin in both conditions. Dark-red and dark-blue dots are genes showing a reduced log2(FC) in response to cisplatin in the *sic-Jun* condition [*i.e*. genes that are less induced (dark red) or repressed (dark blue) by cisplatin when c-Jun is knocked down]. Light-blue or light-red dots are genes that show an increased log2(FC) in response to cisplatin in the *sic-Jun* conditions [*i.e*. genes that are more induced (light red) or more repressed (light blue) by cisplatin when c-Jun is knocked down]. (**C**) Dot-plot showing the inclusion level differences (ILD) following cisplatin treatment in MCF-7 cells transfected with control (*siCtl*, X-axis) or c-Jun (*sic-Jun*, Y-axis) siRNA. All a-SE events that are significant (ILD ≥ 0.1 or ≤–0.1, FDR < 0.05 and TPM ≥ 1) in the *siCtl* condition are considered. Black dots are a-SE with reduced ILD in response to cisplatin in the *sic-Jun* condition (*e.g*. a-SE whose change induced by cisplatin is less pronounced in *sic-Jun*-treated cells compared to *siCtl* cells). Light grey dots are a-SE with increased ILD in response to cisplatin in the *sic-Jun* condition. (**D**) same as in (C) but for Cisplatin-/RBM39-dependent (left panel) or Cisplatin-/RBM39-independent a-SE (right panel). Linear regression lines and equations are shown. (**E**) RBM39-dependent a-SE (see Figure 4F) in *SLC37A4* (exclusion of E7) (*n* = 4), *PCED1A* (exclusion of E7) (*n* = 4), *AMZ2* (exclusion of E3) (*n* = 3) and ACIN1 (exclusion of E4) (n = 3) were selected for validation by RT-PCR. SOR were quantified for each a-SE in MCF-7 cells transfected with a control (*siCtl*) or one of two independent siRNA against c-Jun (*sic-Jun#1* or *sic-Jun#2*) and treated (CIS) or not (NT) with cisplatin (50 μM, 24 h). Histograms are means ± SD of SOR quantified from three or four independent experiments as indicated. Statistical significance was calculated by Two-Way ANOVA (ns: not significant, *P< 0.05, **P< 0.01, ***P< 0.001, ****P < 0.0001). (**F**) Overlap between RBM39-/cisplatin-dependent a-SE in MCF-7 and c-Jun-regulated genes. Statistical significance was calculated by Fisher exact test. ns: not significant. (**G**) Venn diagram showing the non-significant overlap between RBM39-/cisplatin-dependent a-SE and phospho-c-Jun-bound genes after cisplatin treatment (from ChIP-Seq analysis in BT474 (66)]. Statistical significance was calculated by Fisher exact test. ns: not significant. (**H**) Visualisation of ChIP-Seq peaks in control (IgG Ctl IP, green trace) and c-Jun (c-Jun IP, blue trace) ChIP-Seq experiments (two replicates, n1 and n2). Red boxes indicate lack of specific c-Jun binding in the promoter or around E4 and E5 of *COASY*.

To extend our findings beyond COASY, we evaluated the importance of c-Jun in genome-wide cisplatin-mediated AS by performing RNA-seq analysis on c-Jun-depleted MCF-7 cells (*n* = 3) and comparing their transcriptomic response to cisplatin to that of control cells (Supplementary Figure S6A and Supplementary Tables S5,6). In agreement with its hypo-phosphorylated status in basal conditions (Figure 5C) knocking down c-Jun led to relatively limited changes in mRNA levels as we identified only 309 differentially expressed RNA between *siCtl* and *sic-Jun* cells (Supplementary Table S5). The majority of these were mRNAs (85%, 262/309) that were slightly more down- (55%, 144/262) than up-regulated (45%, 118/262). As expected, a GSEA of these modulated mRNA against ‘C3 transcription factors targets collection’ (47) revealed a negative enrichment score for several AP-1-related C3 TF lists in cells depleted for c-Jun (Supplementary Figure 6B). Remarkably, the depletion of c-Jun had a more pronounced effect on AS. Using our analytical pipeline, we identified a total of 1,012 AS events (FDR < 0.05, |ILD| ≥ 0.1 and TPM ≥ 1 in one condition), among which cassette exons were largely dominant (71.3%, 722/1,012) and slightly tilted towards decreased (59.3%,428/722) than increased (40.7%, 294/722) inclusion (Supplementary Figure 6C).

c-Jun depletion significantly attenuated the transcriptomic responses of MCF-7 towards cisplatin. At the steady-state mRNA levels, although the sets of genes that were differentially regulated by cisplatin in *siCtl*- and *sic-Jun*-treated cells largely overlapped, c-Jun knockdown reduced the total number of cisplatin-modulated mRNA by approximately 38% [from 1832 (872 + 960) DE mRNA to 1146 (960 + 183) DE mRNA in *siCtl* and *sic-Jun* treated-cells, respectively] (Figure 6A). When examining the 960 transcripts that were still affected by cisplatin in *sic-Jun*-treated cells, we observed that for 80% of them, the effect of cisplatin was globally slightly less pronounced in the absence of c-Jun on a per gene basis (*e.g*. 50.8% of genes were less repressed and 29.8% of genes were less induced by cisplatin in the absence of c-Jun) (Figure 6B). The knockdown of c-Jun had even a stronger effect on cisplatin-induced AS (Figure 6C and Supplementary Table S6). Depletion of c-Jun reduced the total number of detected cisplatin-induced a-SE [with FDR < 0.05, |ILD| ≥ 0.1 and TPM ≥ 1 in one condition (NT or CIS)] by approximately 2-fold, from 2045 in *siCtl*- to 1042 in *sic-Jun*-treated cells (compare Figure 1D with Supplementary Figure S6D). The amplitude of the splicing changes induced by cisplatin in *siCtl*-treated cells was nearly systematically reduced in *sic-Jun*-treated cells (Figure 6C). The importance of c-Jun in the splicing response induced by cisplatin was more pronounced when looking specifically at the RBM39-dependent vs RBM39-independent a-SE (Figure 6D). Because the effects of cisplatin were more reduced in the absence of c-Jun, these a-SE define a set of splicing changes that rely on c-Jun-mediated inactivation of RBM39 upon cisplatin treatment. The reduction of the effects of cisplatin by c-Jun KD was validated on representative RBM39-dependent a-SE by RT-PCR (Figure 6E).

Interestingly, the effect of cisplatin on skipping of E4–5 of *COASY* and other representative c-Jun/RBM39-dependent a-SE was unaffected by the JNK-IN-8 inhibitor (Supplementary Figure S6E). This is consistent with our observation that the interaction between RBM39 and c-Jun is still induced by cisplatin, even in the presence of JNK-IN-8 and indicates that the role of c-Jun in cisplatin-mediated splicing changes is independent of its transcriptional activity (Figure 5D). To test this on a more global scale, we used several approaches to evaluate whether the a-SE regulated by the cisplatin/c-Jun/RBM39 axis affected preferentially mRNA transcribed from c-Jun target genes. First, we compared the corresponding genes (*n* = 468) against the C3 transcription factor target gene set from the Molecular Signatures Database (MSigDB) and found no significant overlap for AP-1 related gene set. We then built our own set of 306 experimentally verified AP-1 dependent genes defined as the interaction between two publicly available lists, TREDD (64) and TRRUST (65). There were only seven genes in common between these high-confidence AP-1 targets and the 468 alternatively-spliced genes, which did not represent a significant overlap (Figure 6F). We also interrogated the set of alternatively spliced genes against a published list of 209 different promoters bound by c-Jun after cisplatin treatment in the BT474 breast cancer cell and found no significant overlap (66) (Figure 6G). Finally, analysis of publicly available ChIP-seq datasets for c-Jun in MCF-7 (ENCLB508LER from the ENCODE project) revealed no significant enrichment of c-Jun peaks around the TSS of differentially spliced genes or 250 bp around a-SE as illustrated for COASY (Figure 6H). Altogether, these results suggest that c-Jun participates in cisplatin-dependent splicing, independently of its transcriptional function and of its presence on DNA.

## DISCUSSION

The proximal DDR response relies on intracellular signalling pathways that culminate into the reprogramming of cellular gene expression. Originally, research in the field focused on the quantitative transcriptomic changes (*i.e*. genes that are up- or down-regulated) associated with TFs such as p53 and NF-κB that coordinate expression of specific sets of genes (28,67,68). More recently, it was found that the DDR response also imposes qualitative changes on the cellular transcriptome. In particular, DDR drives the production of specific splicing isoforms of functionally related mRNA (33,34). A series of important questions stemmed from these findings: (i) how do these alternatively spliced isoforms contribute to the DDR response, (ii) what are the splicing factors responsible for DDR-induced AS and (iii) how does DDR signalling impact the activity of these splicing regulators. Our study provides important elements that partially answer these questions.

Large-scale transcriptome analyses have identified transcripts whose splicing is affected by genotoxic treatments. It was established that these transcripts often code for proteins that could participate in the DDR, such as cell-cycle and apoptosis regulators, chromatin modifiers, and gene expression regulators (69–71). In this study, we identified transcriptome-wide changes in AS associated with cisplatin in breast cancer cells. In agreement with previous reports, we found that cisplatin-induced AS affects specific functional classes of mRNA, including mRNA coding for proteins involved in DNA repair and replication, cell division as well as mRNA synthesis and processing, all of which are intimately linked to the DDR (32,72–74). Interestingly, these functional classes are different from those associated with genes that are differentially expressed in response to cisplatin, supporting the idea that control of alternative pre-mRNA splicing constitutes a separate arm of the DDR response that could actively participate in the management of the damage and influence cell fate (33). Among cisplatin-induced AS events, we identified exclusion of E4–5 of *COASY* as being a major contributor to sensitivity to cisplatin in breast cancer cells. COASY is a bi-functional enzyme that catalyses the last two steps of Coenzyme A biosynthesis and is found in the mitochondrial matrix (37). Beyond cell cycle, apoptosis and gene expression, our study thus extends the functional repertoire of transcripts affected by cisplatin-induced AS to those involved in mitochondrial functions. Exons 4 and 5 encode amino-acids 350 to 425 of COASY, which correspond to approximately the first third of its dephospho-CoA kinase (d-PCoAK) domain, including the ATP-binding pocket. Because the d-PCoAK domain catalyses the ATP-dependent phosphorylation of 3'-dephospho-CoA into CoA, a COASY isoform lacking exons 4 and 5 would be expected to be inactive and reduce CoA production by the mitochondria. Impaired CoA synthesis has been associated with mitochondrial dysfunction (75). For instance, mitochondria from mice knocked-down for pantothenate-kinase 2 (PANK2), the first enzyme in the CoA biosynthetic pathway, or for COASY exhibit morphological and functional defects (38,76,77). While the underlying mechanism remains unclear, our finding that overexpression of the COASY- short isoform affects mitochondrial dynamics strengthens the idea that defective CoA biosynthesis leads to mitochondrial dysfunction. Multiple studies have reported that cisplatin exposure results in mitochondrial injury (78–80). To date, the mechanism of cisplatin-induced mitochondrial impairment is thought to result from damage to mtDNA, which reduces expression of mitochondrial proteins leading to dysfunctional mitochondria, independently of the effects of cisplatin on nuclear DNA (nDNA) (22,57). Our study suggests that cisplatin-induced changes in alternative splicing of nDNA-encoded pre-mRNAs also contribute to its effects on mitochondrial functions. Overall, our observations point towards a model in which expression of the COASY-short isoform and associated mitochondrial dysfunction participates in cisplatin-induced apoptosis. Indeed, overexpression of COASY-short isoform affects mitochondrial fusion and leads to a ‘tubular’ phenotype similar to that observed upon treatment with cisplatin. In addition, preventing cisplatin-induced expression of COASY lacking E4–5 reduces the effects of cisplatin on mitochondria and prevents cell death. While mitochondrial fusion is normally induced by cisplatin, it does not occur when expression of COASY-short isoform is prevented. Mitochondrial fusion and increased oxidative capacity are usually considered as compensatory mechanisms, which help maintaining energy output in face of cellular stress (24). It is therefore surprising that reducing the expression of COASY-short isoform (and thus preventing mitochondrial fusion) is beneficial to cisplatin-treated cells and increases their resistance to cisplatin. However, our findings are fully consistent with several recent publications suggesting that the cytotoxic effects of cisplatin are mediated by its ability to promote mitochondrial fusion and induce an oxidative stress response (22,57,81). We propose that in the absence of COASY-short, cisplatin-mediated cytotoxicity linked to mitochondrial activity is reduced because of dysfunctional mitochondria. Our results thus identify COASY-short as an attractive therapeutic target to improve the effectiveness and/or reduce the toxicity of cisplatin-based chemotherapies. Because the COASY-short isoform is also induced by treatment with camptothecin and doxorubicin (Figure 2D), our findings might also offer benefits in the context of combination chemotherapies.

To date, the pathways and molecular players that orchestrate DDR-induced splicing changes remain poorly characterized (31,82). Identification of the AS event in the *COASY* pre-mRNA, gave us the opportunity to look for upstream effectors mediating the effects of cisplatin on pre-mRNA splicing. Using COASY as a paradigm, we undertook a siRNA screen and identified a series of RNA-binding proteins (RBPs) whose knockdown modified inclusion of E4–5 of *COASY*. Interestingly, the siRNAs with the largest effects (*i.e*. ΔΨ>20%) all led to a reduction in E4–5 inclusion, similarly to cisplatin. The majority of these siRNA were directed against members of the core spliceosome, including subunits of the SF3A (*i.e*. SF3A1, -2 and -3), SF3B (*i.e*. SF3B4) and U2 auxiliary factor (U2AF) (*i.e*. U2AF1) complexes, all of which are associated with U2 snRNP, and PRPF8, a key spliceosome assembly factor. Three notable exceptions were i) SR-A1 [a.k.a, SCAF1, Serine/Arginine Rich (SR)-related C-terminal domain-associated factor 1] a poorly characterized SR family protein, ii) MBNL1 (Muscleblind-like 1), a RBP known for its role in muscular dystrophy and, (iii) RBM39. RBM39 is a U2AF-related splicing factor that also plays a role in transcriptional regulation and translation (10,83). Functional connections between RBM39 and U2AF have been previously described (84,85). Therefore, it is not surprising that they were both identified in our siRNA screen. In addition, physical interactions between RBM39 and SF3A1/2/3, SF3B4, PRPF8 and MBNL1 have also been reported (Figure 5A) (10,83). These results suggest that RBM39 controls splicing of E4–5 of COASY as part of a large network of splicing factors. Along these lines, it would be interesting to test a potential functional collaboration between RBM39 and other RBPs identified in our screen, including AS-R1 and MBLN1. The network of interactions between RBM39, core spliceosome components and other RBPs as well as the dynamics of these interactions are interesting issues that deserve to be investigated further.

RBM39 is upregulated in most cancers and its inhibition is lethal or cytostatic in several cellular models of breast cancers (86). Although these observations have fuelled the idea that inhibiting RBM39 might be a promising therapeutic opportunity for breast cancers, the exact function of RBM39 in cancer in general and in breast cancer in particular remains unclear (15). In triple negative breast cancer cells (TNBC), the pro-apoptotic effect of RBM39 depletion was attributed to transcriptional effects (86). In MCF-7 cells, depletion of RBM39 was associated with downregulation of cell cycle regulators (87). Therefore, the importance of RBM39 in breast cancer biology has been mostly linked to its role in transcriptional regulation. Our results also support the idea that the splicing regulatory activity of RBM39 is important for breast cancer cell biology and adaptive response towards cisplatin.

In agreement with previous observations in other cell types, knockdown of RBM39 led to a large number of splicing changes in MCF-7 cells, the vast majority of which affecting cassette exons (SE) (10). Comparison with cisplatin-dependent a-SE showed a highly significant overlap between both data sets, with almost 30% of cisplatin-dependent a-SE also being affected by RBM39 knockdown. Strikingly, these common a-SE were almost entirely similarly affected by cisplatin or RBM39 knockdown. These observations support the model that an important fraction of cisplatin-induced a-SE involves inactivation of RBM39, as illustrated for *COASY* E4–5. Our findings also raise two corollary issues. First, not all cisplatin-induced a-SE are found in the set of RBM39-dependent events, suggesting that other RBPs might be involved in mediating the effects of cisplatin on AS. In addition, whereas RBM39 knockdown recapitulates a large proportion of the effects of cisplatin on SE, only a small fraction of RBM39-dependent a-SE events (12%, 577/4893) are sensitive to cisplatin. Several non-exclusive mechanisms could underlie this specificity. For instance, cisplatin-mediated inactivation of RBM39 could occur only at specific cassette exons. Alternatively, RBM39- and cisplatin-dependent exons could be those that are the most sensitive to RBM39 presence or those whose splicing relies on multiple cisplatin-sensitive RBPs, in addition to RBM39. Along these lines, combinatorial control of alternative splicing by multiple RBPs during oxaliplatin-induced DDR has been reported (82). The issue of cisplatin specificity towards alternative splicing is undoubtedly an important one, that deserves to be investigated further.

One of the most unexpected findings in this study relates to the mechanism by which cisplatin promotes inactivation of RBM39 splicing activity. Based on a limited number of published examples, the emerging trend is that the DDR achieves reprogramming of the splicing landscape by promoting post-translational modifications of splicing factors or modifying their expression levels or intracellular localisation (33). In our case, RBM39 levels or subcellular localisation remained unchanged after cisplatin treatment. Our previous results indicated that canonical ATM and ATR kinase were not involved in cisplatin-induced changes in splicing in MCF-7 (32). Instead, we identified a novel regulatory mechanism that involves a physical interaction between RBM39 and the transcription factor c-Jun. The interaction between RBM39 and c-Jun has been reported by others and is thought to be important for RBM39 to act as a co-activator of the AP-1 transcriptional complex (16). Here, we shed light on the other side of the coin and show that by binding to RBM39, c-Jun prevents its association with target pre-mRNAs and inhibits its splicing activity. Targeting the association between RBM39 and c-Jun was proposed as an attractive therapeutic opportunity for breast cancer (89). However, our results now show that such an option might not only impact on AP1-regulated transcriptional programs, as previously thought, but will also most likely impact RBM39-controlled splicing processes.

**Figure 7.**
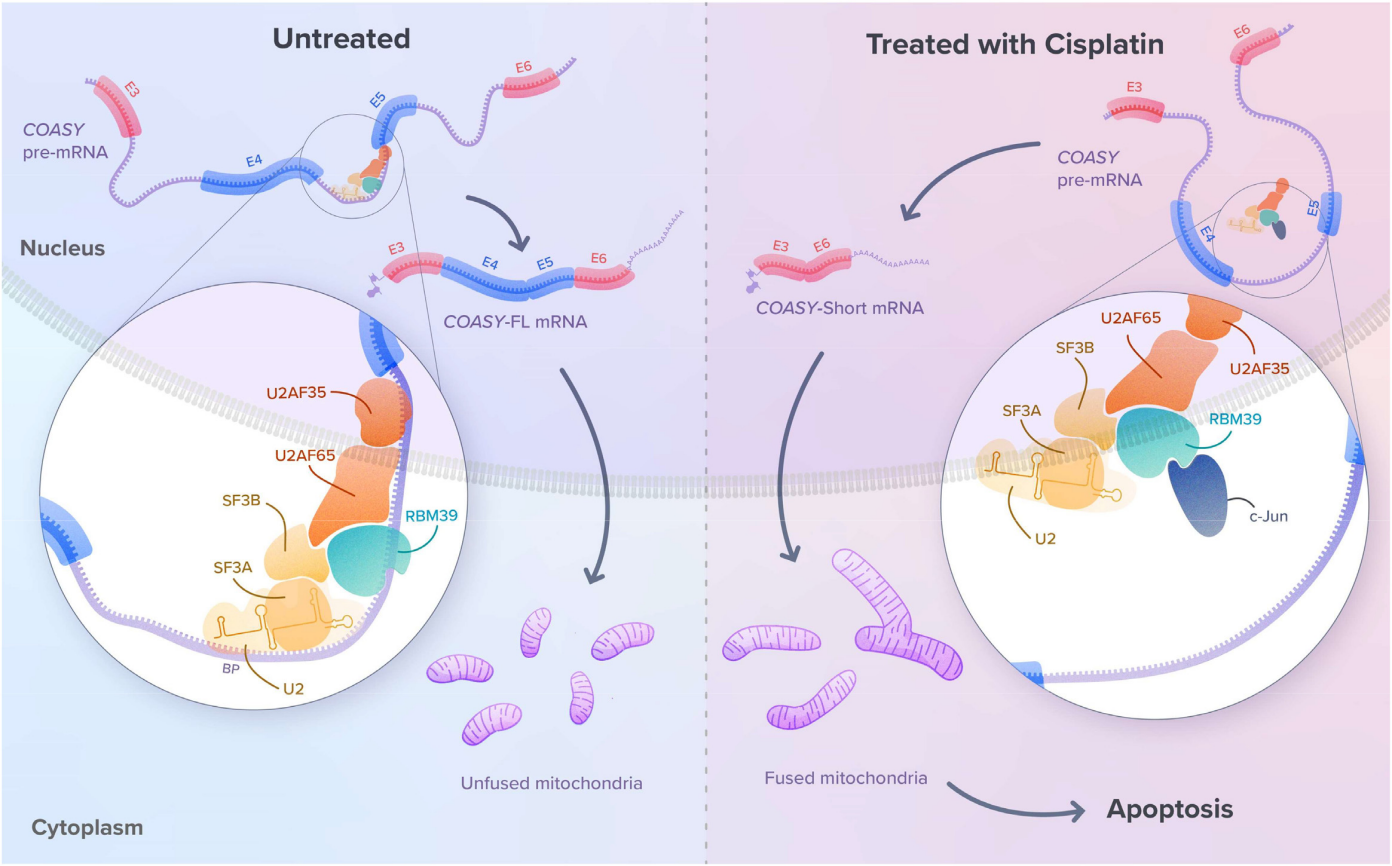
Model for regulation of *COASY* pre-mRNA splicing by c-Jun in response to cisplatin. Treatment with cisplatin promotes the interaction between c-Jun and RBM39, that prevents binding of RBM39 to intron 4 of *COASY* pre-mRNA. This leads to expression of the *COASY*-short isoform, which promotes mitochondrial fusion and apoptosis.

Evidence for an involvement of TF in alternative splicing has been accumulating in recent years. In addition to the obvious situation where a TF controls splicing indirectly by increasing or decreasing the expression of splicing factors (SF), more sophisticated modes of action are being described. For instance, TFs control splicing by interfering with RNA polymerase II elongation (‘kinetics’ model), by recruiting splicing effectors to sites of transcription (‘promoter or gene-body recruitment’ models) or more recently, by actively participating in splicing complexes (4,7,90,91). Here, we document yet another mode of action, as we show that by associating with RBM39, c-Jun prevents its binding to *COASY* pre-mRNA (Figure 7). This model of a TF binding to and inhibiting a SF might typify other reported TF-SF interactions (7). In addition to c-Jun, RBM39 interacts with other TFs, including MYC (92), p53 (93) and ESR1/2 (16). In light of our findings, a regulation of RBM39 splicing activity by other TFs deserves to be tested. In MCF-7 cells, knocking down c-Jun leads to very moderate effects on mRNA steady-states levels. This is consistent with the observation that c-Jun is transcriptionally inactive in the absence of cisplatin, because of reduced phosphorylation of its TAD. Remarkably, downregulation of c-Jun correlated with a large set of AS events in unstimulated cells, suggesting that c-Jun might have additional roles in splicing regulation that are independent of cisplatin.

How c-Jun promotes dissociation of RBM39 from its target pre-mRNA remains to be characterized. RBM39 has two central canonical RRM motifs (RRM1 from aa 153–230 and RRM2, from aa 250–328) and one U2AF homology motif (UHM; aa445-508) in its C-terminal region (15). The UHM is important for the interaction with U2AF65 (85). While a first report indicated that c-Jun interacts with aa 291–406 of RBM39, a region partially overlapping with RRM2 (16), a second study identified an additional c-Jun interaction interface within the C-terminal UHM domain (88). Based on these findings, one could assume that interaction with c-Jun could hinder binding of its RRM2 to RNA and/or its association with U2AF1. With regards to the latter, association with c-Jun would impede the formation of RBM39-U2AF65 liquid-like assemblies, thus hindering the recruitment of U2 snRNP and spliceosome assembly (84). Finally, our findings add to the growing body of evidence implicating TFs in splicing regulation and suggest that beyond transcription, their involvement in other gene expression processes might have to be re-evaluated.

Our study uncovers an entirely novel pathway and its associated molecular effectors that underly the splicing changes downstream of the DDR. Our results also illustrate how reprogramming of splicing processes allows expression of specific spicing isoforms, that functionally contribute to the DDR. In addition to COASY-short isoform, RBM39 and c-Jun, it is likely that other splicing variants, controlled by other SFs and TFs also contribute to DDR-related processes. Identification of these pathways in the context of oncogenic processes or drug resistance should allow the development of better therapeutic molecules integrating the multiple layers of gene regulation.

## DATA AVAILABILITY

RNA-seq data generated in this study have been deposited to GEO under accession GSE195842: https://www.ncbi.nlm.nih.gov/geo/query/acc.cgi?acc=GSE195842.

## Supplementary Material

gkac1130_Supplemental_Files

## Notes

Present address: Tina O’Grady, Department of Pathology and Laboratory Medicine, Tulane University, School of Medicine, New Orleans, LA, USA.

Present address: Charles Lambert, Diagenode S.A., Liège Science Park, Rue du Bois Saint-Jean 3, 4102 Liège, Belgium.

Present address: Gilles Rademaker, Perera Lab, University of California, San Francisco, CA, USA.
