# Supplementary material for: The transcription factor c-Jun inhibits RBM39 to reprogram pre-mRNA splicing during genotoxic stress
Source: Nucleic Acids Res. 2022 Dec 8;50(22):12768–89. doi: 10.1093/nar/gkac1130 (PMC9825188; doi:10.1093/nar/gkac1130)
Supplement: gkac1130_Supplemental_Files [file gkac1130_supplemental_files.zip › Supplementary Figures and Legends.pdf]

SUPPLEMENTARY FIGURES and LEGENDS

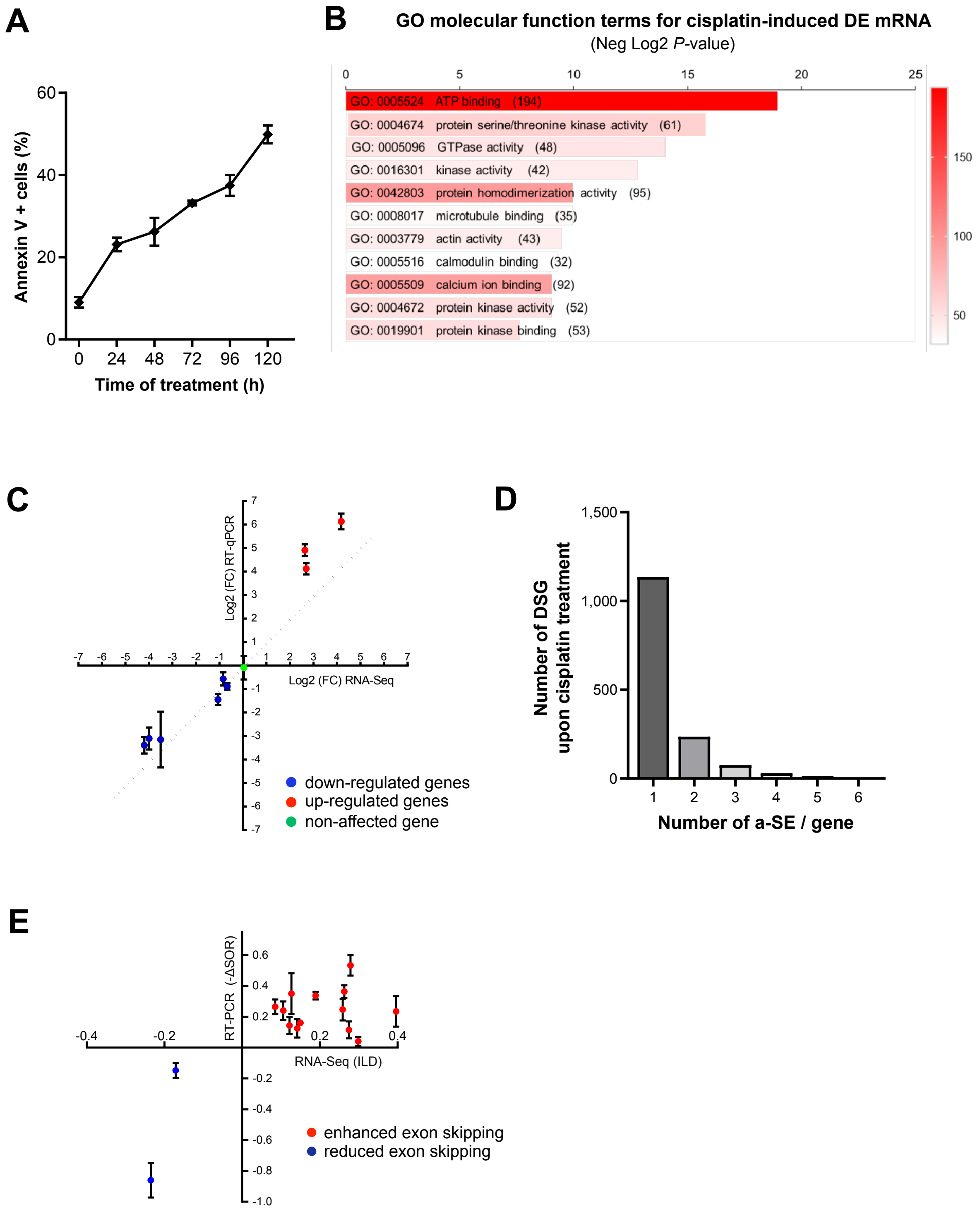

Supplementary Figure S1

Supplementary Figure S1

(**A**) Cisplatin-induced apoptosis was measured in MCF-7 cells during 5 days, every 24 h by flow cytometry. The percentage of Annexin V+ cells are indicated. Histograms represent means ± SD from three independent experiments. (**B**) GO molecular function terms enriched in differentially expressed mRNA following cisplatin treatment in MCF-7 cells. Number of genes associated with each GO term is indicated in brackets. (**C**) Correlation between RNA levels quantified by RNA-seq or RT-qPCR for 10 transcripts (9 mRNAs et 1 lincRNA) expected to be up- (red dots, positive Log2 (FC), adjusted *P*-value <0.05, and TPM ≥1 in at least one experimental condition), down- (blue dots), or not regulated (black dot) following cisplatin treatment in MCF-7 cells. Results from RT-qPCR are presented as mean ± SD from three independent experiments. (**D**) Distribution of DSG (mRNA) according to the number of cisplatin-induced a-SE they are affected with. (**E**) Fifteen cisplatin-induced a-SE identified in the RNA-seq were selected for validation by end-point RT-PCR. Red dots: positive ILD ≥0.1 corresponding to an increase in exon skipping in response to cisplatin with an adjusted *P*-value <0.05 and TPM >20. Blue dots: negative ILD ≤-0.1 corresponding to a reduction in exon skipping in response to cisplatin with an adjusted *P*-value <0.05 and TPM >20. The cisplatin-induced difference between spliced-out ratios SOR (short/FL isoforms) is reported on Y-axis. Results are average of three replicates and presented as mean ± SD.

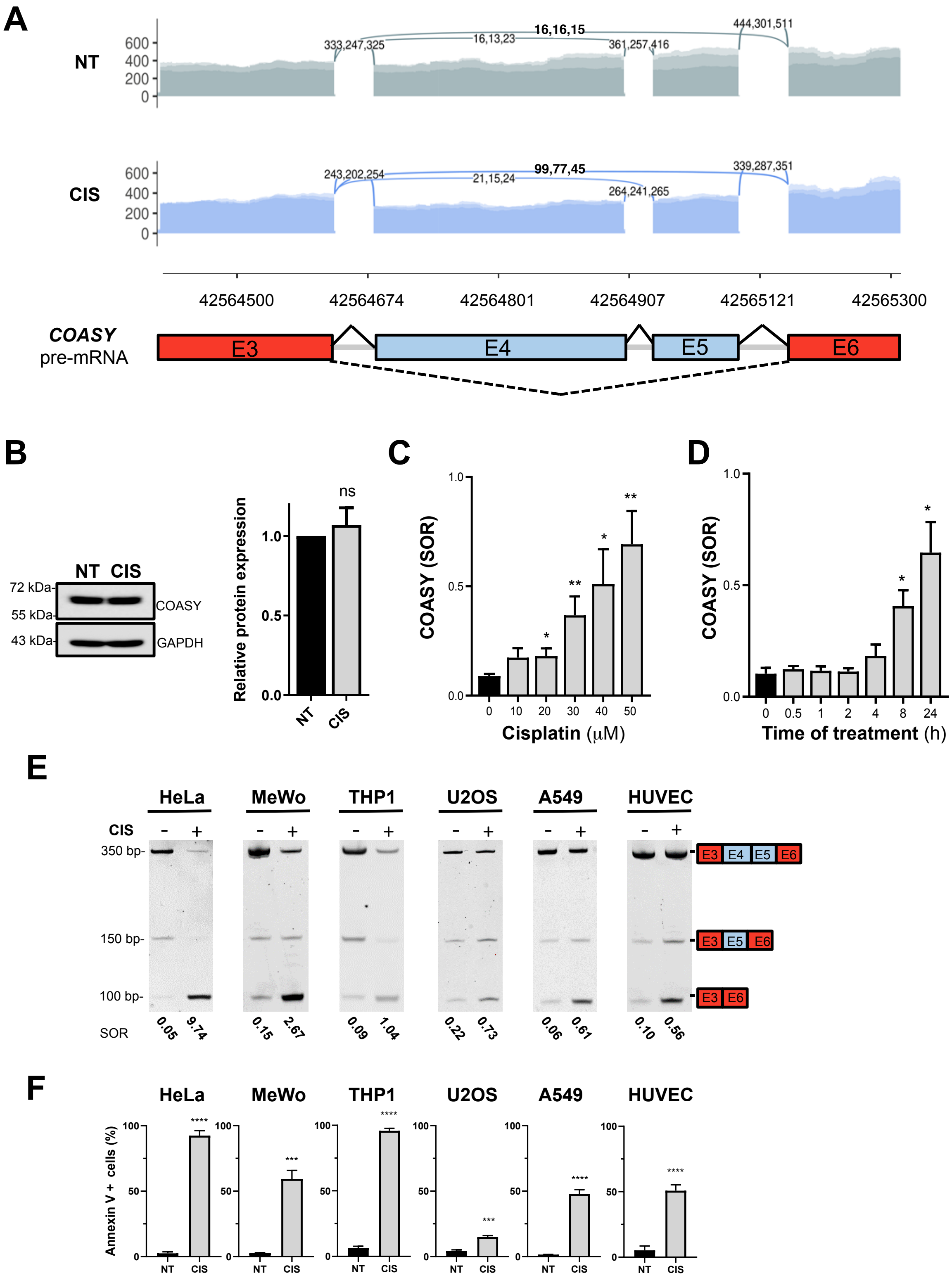

Supplementary Figure S2

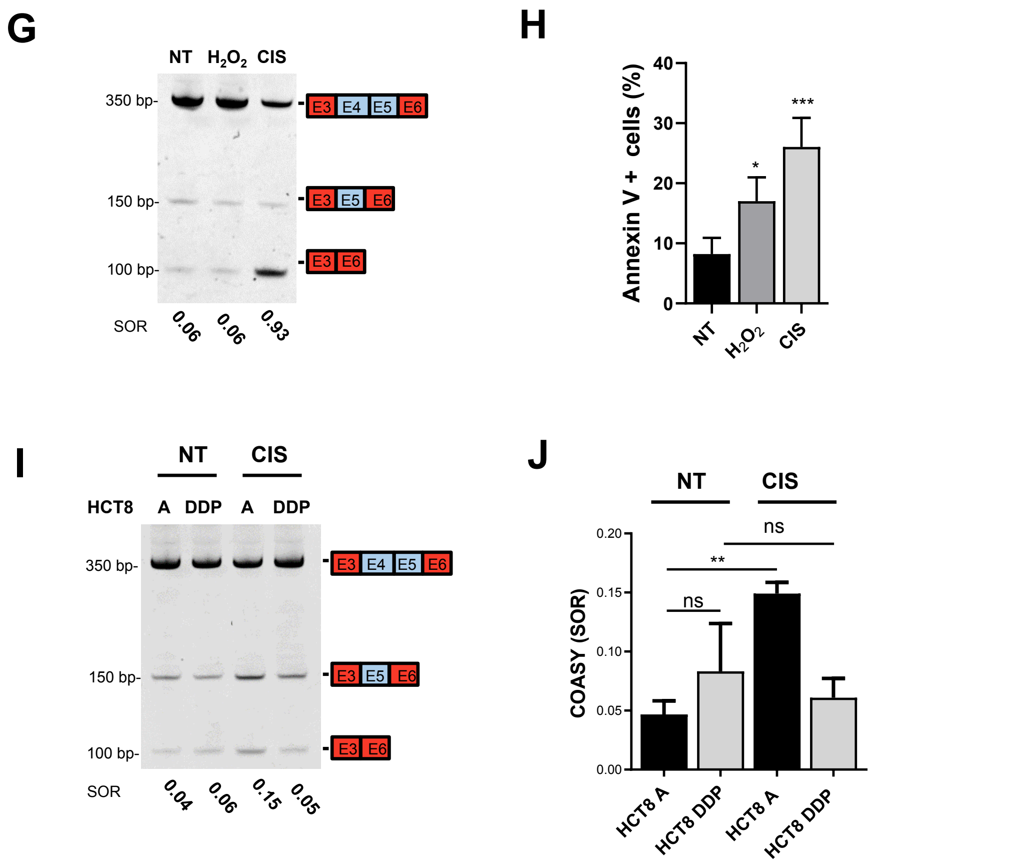

Supplementary Figure S2 continued

Supplementary Figure S2

(**A**) Sashimi plot of *COASY* transcript from E3 to E6**.** The blue trace represents the cisplatin-treated condition, and the grey one represents the non treated control condition. X-axis indicates genomic locations on chromosome 17(+) strand, and the Y-axis indicates transcription intensity (number of RNA-seq reads). The numbers above the bridges indicate the number of junction-spanning reads. Reads corresponding to skipping of exons 4 and 5 are indicated in bold. (**B)** Representative immunoblots of COASY in MCF7 cells treated or not with cisplatin (50 µM, 24h) (left) and quantification of COASY/GAPDH ratio (right) based on 5 independent experiments. Statistical analysis was calculated by Student's t-test. (ns: not significant). (**C-D**) Quantifications of the splicing out ratio (SOR) short/ FL isoform from experiments described in Figure 2B (**C**) and Figure 2C (**D**). Histograms represent means SOR ± SEM from three independent experiments. Statistical significance was calculated by Student's *t*-test (**P*<0.05, ***P*<0.01). (**E**) Detection of FL and short *COASY* isoforms in the indicated cancer cell lines (HeLa, MeWo, THP1, U2OS and A549) and in primary human endothelial cells (HUVEC) treated with cisplatin (50 µM) for 24 h. RNA was extracted, reverse-transcribed and amplified by end-point PCR. Amplification products were discriminated by gel

electrophoresis. The numbers below the gel indicate the spliced-out ratio (SOR) short/FL isoforms. (**F**) Cisplatin-induced apoptosis was measured in the different tumour cell lines by flow cytometry. Percentage of Annexin V+ cells are shown. Means ± SD were determined on 3 independent experiments. Statistical significance was calculated by student's *t*-Test. (****P*<0.001, *****P*<0.0001). (**G**) Detection of FL and short *COASY* isoforms was performed by end-point RT-PCR in MCF-7 untreated (NT) or treated with H_2O_2 (500 µM, 24 h) or cisplatin (50 µM, 24 h). RNA was extracted, reverse-transcribed and amplified by end-point PCR. Amplification products were analyzed by gel electrophoresis. The numbers below the gel indicate the spliced-out ratio (SOR). (**H**) Apoptosis of MCF7 cells treated as in (G) was measured by flow cytometry. Percentage of Annexin V+ cells are shown. Means ± SD were determined on 3 independent experiments. Statistical significance was calculated by student's *t*-Test. (**P*<0.05, ****P*<0.001). (**I-J**) Detection of FL and short *COASY* isoforms was performed by end-point RT-PCR in two isogenic human colon carcinoma cell lines (HCT8) sensitive (HCT8 A) or resistant (HCT8 DDP) to cisplatin before (NT) or after (CIS) treatment with cisplatin (50 µM, 24 h). A representative image of electrophoresis gel analysis of amplicons (**I**) and quantifications (**J**) from three independent experiments are shown. Histograms represent means SOR ± SD. Statistical significance was calculated by Two-Way ANOVA and Bonferroni's multiple comparison test (n=3, ns: not significant, ***P*<0.01).

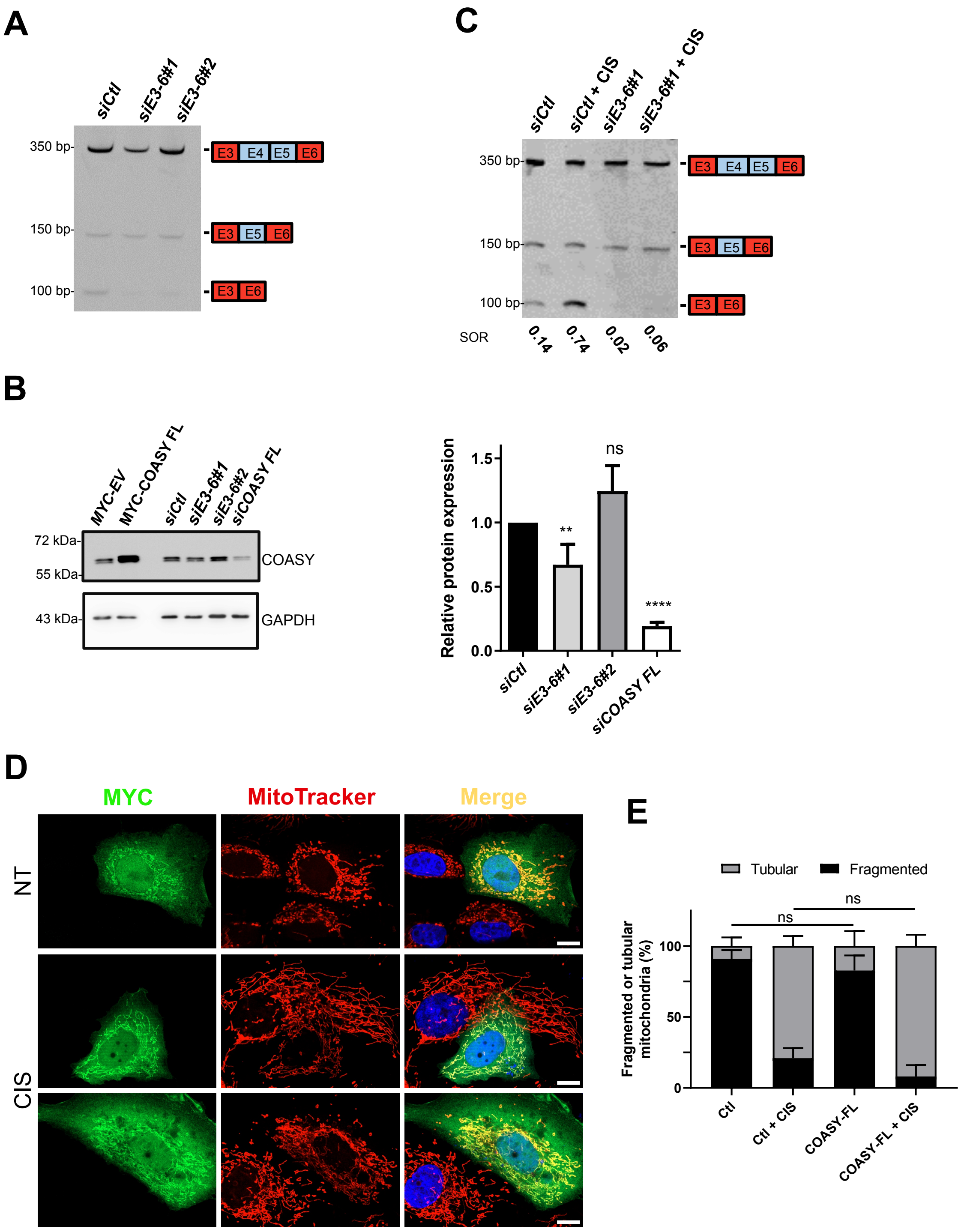

Supplementary Figure S3

Supplementary Figure S3

(**A**) Validation by end-point RT-PCR of knockdown of *COASY short isoform* by specific siRNAs targeting the junction between exon 3 and exon 6, *siE3-6#1* and #2. MCF-7 cells were transfected with

the indicated siRNAs and 48 h post-transfection, RNA was extracted, reverse-transcribed and amplified by end-point PCR. Amplification products were discriminated by gel electrophoresis. Image is representative of multiple independent experiments. (**B**) Left: Representative Immunoblot blot analysis of COASY in MCF7 cells transfected with control MYC-empty vector (EV) or MYC-COASY-FL or with siRNAs targeting *COASY* short (*siE3-6#1* or *#2*) or long (*siCOASY FL*) isoforms. GAPDH (loading control). Right: Quantification of COASY/GAPDH ratio was performed with Image-Quant and is shown relative to the *siCtl* condition. Histograms represent means ± SD from five independent experiments. Statistical analysis was calculated by Student's t-Test (ns: non significant, ***P*<0.001, *****P*<0.0001). (**C**) Detection of FL and short *COASY* isoforms was performed by end-point RT-PCR in MCF-7 transfected with control siRNA (*siCtl*) or an siRNA specifically targeting the *COASY* short isoform (*siE3-6#1*) and treated (CIS) or not (NT) with cisplatin (50 µM, 24 h). Numbers below the gel indicate the spliced-out ratio (SOR) short/FL isoforms. Image is representative of three independent experiments. (**D**) Immunofluorescence images of MYC-COASY full-length (in green) and mitochondria staining by Mito Tracker (in red) in MCF-7 cells transiently transfected. Nuclei are stained in blue by Hoechst 33342. Scale bar: 10 µM. (**E**) Quantification of percentage of cells with tubular or fragmented mitochondrial network observed in (D) (n=50).

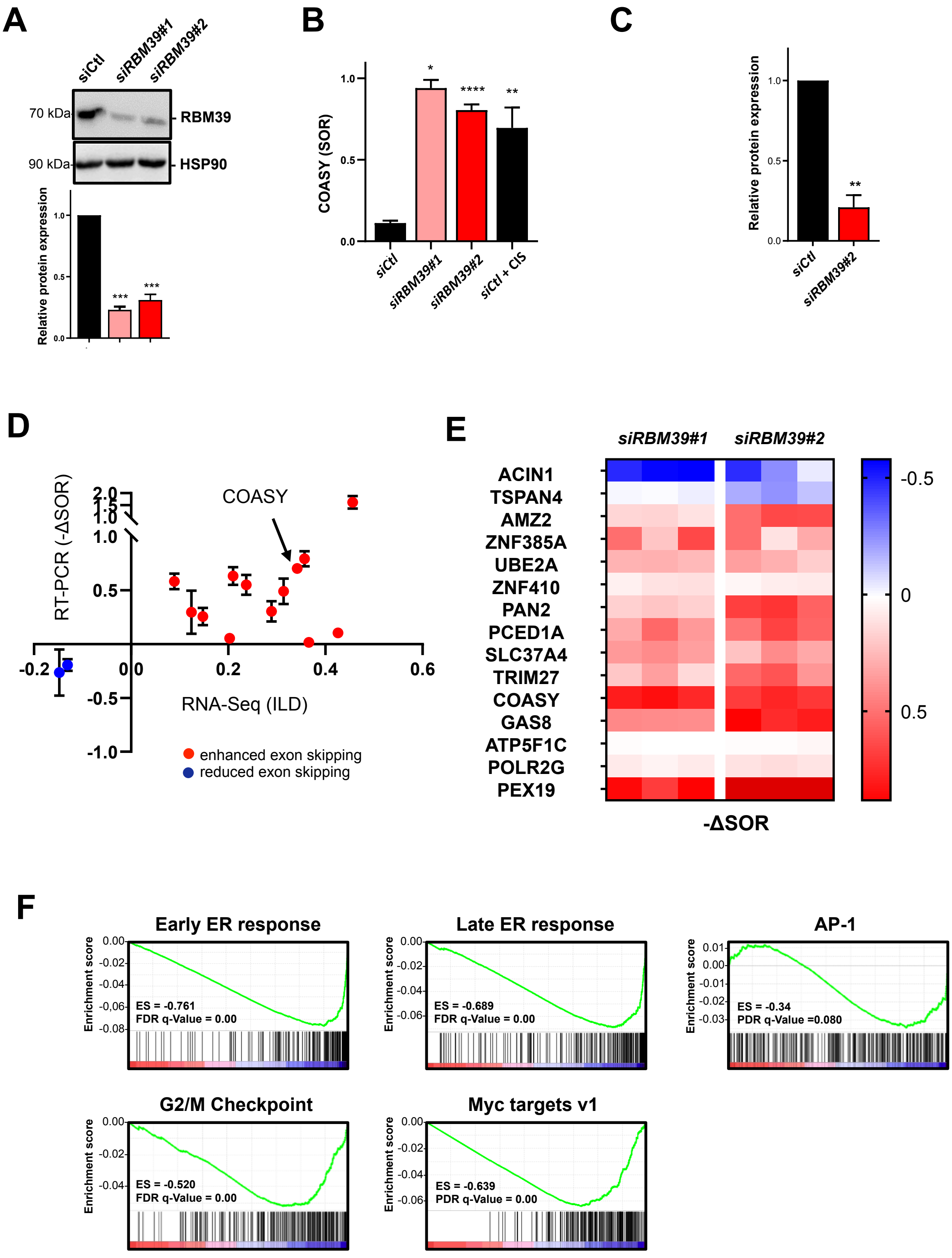

Supplementary Figure S4

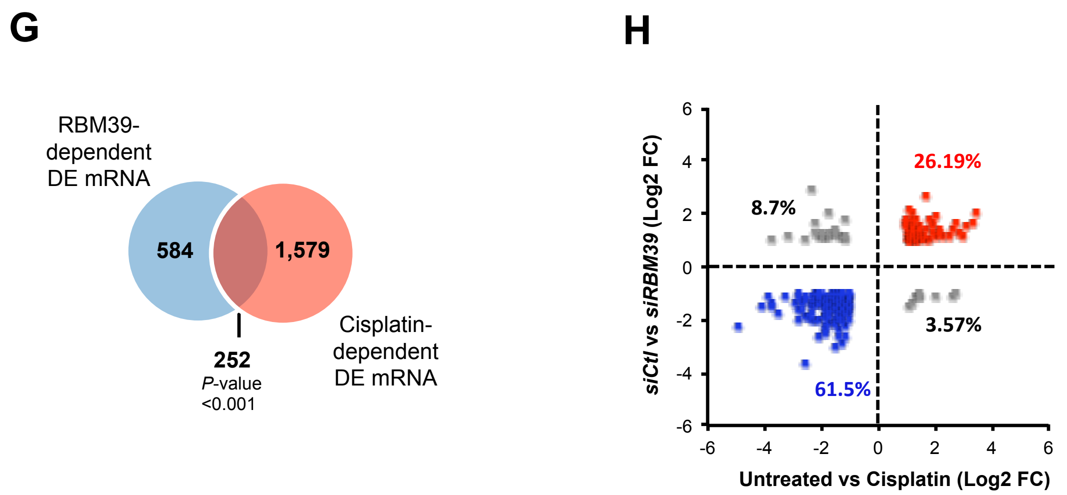

Supplementary Figure S4 continued

Supplementary Figure S4

(**A**) Top: Western blotting of RBM39 in MCF-7 cells transfected with control (*siCtl*) or independent RBM39-specific siRNAs (*siRBM39#1* or *siRBM39#2*). HSP90 was used as a loading control. Bottom: Quantifications of the RBM39/HSP90 ratio was performed with ImageQuant and is expressed relatively to the *siCtl* condition. Histograms represent means ± SD from three independent experiments. Statistical significance was calculated by Student's *t*-test (***P<0.001). (**B**) Detection of *COASY* FL or short isoforms was performed by end-point RT-PCR in MCF-7 cells transfected with control siRNA (*siCtl*) or one of two independent *RBM39*-specific siRNA (*siRBM39#1* and *siRBM39#2*) and treated (+CIS) with cisplatin (50 µM, 24 h) when indicated. Quantifications of SOR (short/FL) from three independent experiments are shown. Histograms represent means SOR ± SD. Statistical significance was calculated by Student's *t*-test. (*P<0.05, **P<0.01, ***P<0.001). (**C**) Validation of the RBM39 knockdown in MCF-7 cells used for RNA-seq analysis. Quantifications of the RBM39/HSP90 ratio was performed with ImageQuant after western blotting as in (A) and is expressed relative to the *siCtl* condition. Histograms represent means ± SD from three independent experiments. Statistical significance was calculated by Student's *t*-test (**P<0.01). (**D**) Fifteen RBM39-dependent a-SE identified by RNA-seq in MCF-7 cells transfected with a specific siRNA (*siRBM39#2*) with |ILD| ≥ 0.1 an adjusted *P*-value<0.05 and TPM> 20 were selected for experimental validation by end-point RT-PCR. Red dots represent a-SE characterized by a positive ILD ≥ 0.1, corresponding to an increase in exon skipping in response to RBM39 knockdown. Blue dots represent a-SE characterized by a negative ILD ≤ -0.1, corresponding to a reduction in exon skipping in response to RBM39 knockdown. Experimental validation of selected events was performed on total RNA extracted from MCF-7 cells

transfected with control siRNA (*siCtl*) and a RBM39-specific siRNA (*siRBM39#2*). Difference in SOR between *siRBM39* and *siCtl* conditions is reported on Y-axis. Results are average of three replicates and presented as mean ± SD. (**E**) Heat map of SOR differences in *siRBM39* vs *siCtl* conditions, with two independent RBM39-specific siRNAs (*siRBM39#1* and *siRBM39#2*) measured for the 15 RBM39-dependent a-SE shown in (D). Red and blue values correspond to an increase and decrease in exon skipping, respectively (n=3). (**F**) Top GSEA terms associated with differentially expressed genes in MCF-7 cells following knockdown of RBM39. (**G**) Venn diagram showing the overlap between differentially expressed (DE) mRNA following RBM39 knockdown or cisplatin treatment in MCF-7 cells. P<0.001 by Fisher exact test. (**H**) Correlation between the Log2(FC) in gene expression in response to cisplatin (X-axis) or in response to RBM39 knockdown (Y-axis) of the 252 common DE mRNA shown in (E). The red and blue dots have a Log2(FC)≥1 or ≤-1, respectively in both conditions. Grey dots illustrate DE mRNA with inverse regulation by cisplatin or RBM39 knockdown.

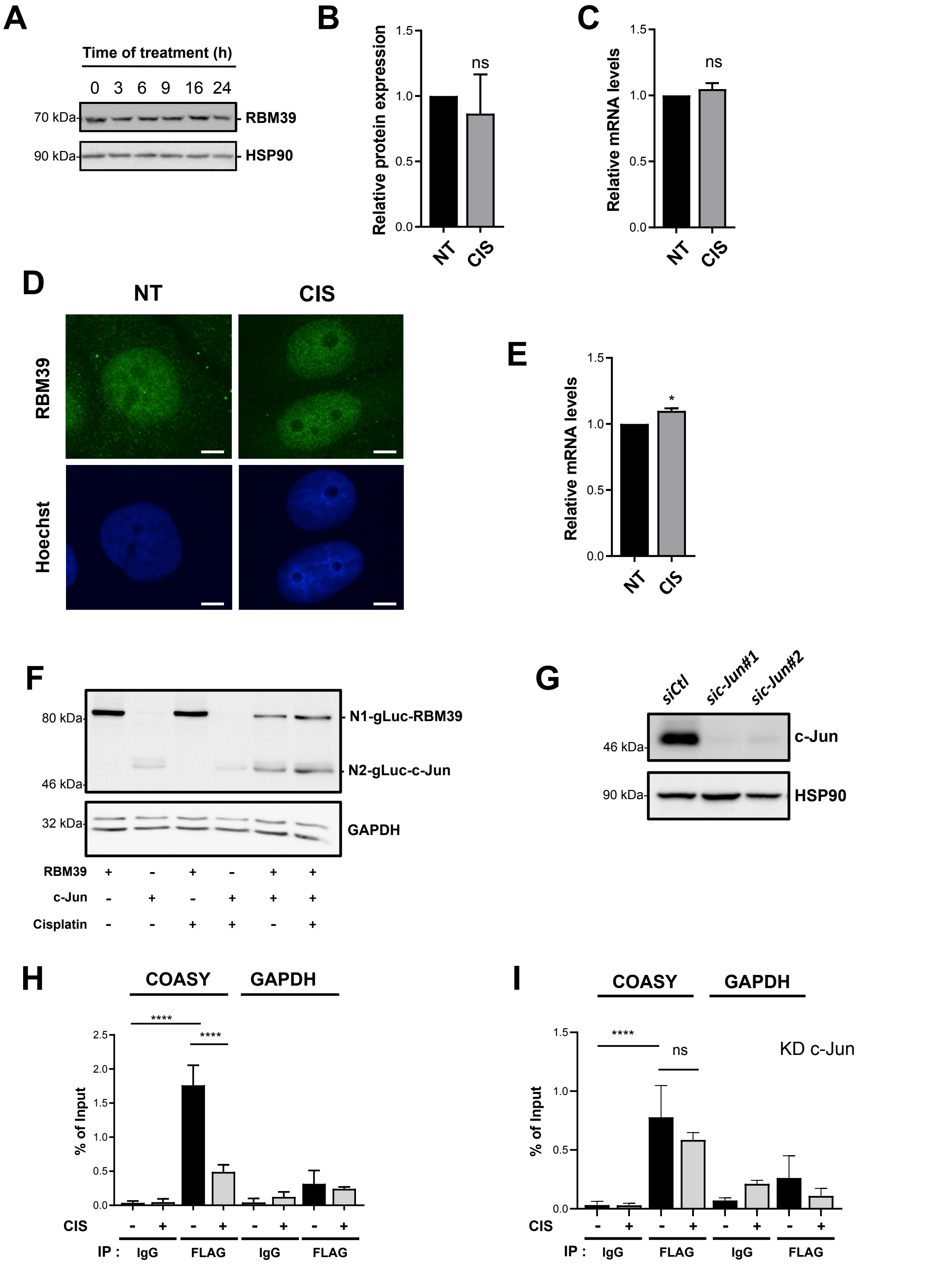

Supplementary Figure S5

Supplementary Figure S5

(**A**) Levels of RBM39 were assessed by immunoblotting on total protein extracts from MCF-7 cells, treated with cisplatin for the indicated times. HSP90 is used as a loading control. (**B**) Quantification of

the RBM39/HSP90 ratio at 24h of cisplatin treatment was performed by signal densitometry analysis from experiments described in (A) and are expressed relatively to the t=0 time point. Histograms represent means ± SD from five independent experiments. Statistical significance was calculated by one-sample t-test (ns: not significant). (**C**) *RBM39* mRNA levels in cells treated with cisplatin (50 µM, 24 h) were assessed by RT-qPCR and expressed relative to non-treated (NT) cells. Histograms represent means ± SD from five independent experiments. Statistical significance was calculated by one-sample t-test (ns: not significant). (**D**) The intracellular localization of RBM39 (green) was assessed by immuno-fluorescence in MCF-7 cells treated (CIS) or not (NT) by cisplatin (50 µM, 24 h). Nuclei are stained with Hoechst (blue). Scale bar: 5 µm. (**E**) *c-Jun* mRNA levels in cells treated with cisplatin (50 µM, 24 h) were assessed by RT-qPCR and expressed relatively to non-treated (NT) cells. Histograms represent means ± SD from three independent experiments. Statistical significance was calculated by one sample t-test (*P<0.05). (**F**) Expression levels of recombinant *g*LUC-N1-RBM39 and *g*LUC-N2-c-Jun used in gPCA experiments described in Figure 5B were assessed with an antibody recognizing both N1 and N2 fragments of the *gaussia* Luciferase. GAPDH is used as a loading control. Image illustrates one representative experiment. (**G**) Western blotting of c-Jun in MCF-7 cells following transfection with control (*siCtl*) or one of two independent *c-Ju*n-specific siRNA (*sic-Jun#1* and *sic-Jun#2*). HSP90 was used as a loading control. Image illustrates one representative experiment used in Figure 5G (and H). (**H**) MCF-7 cells transfected with Flag-RBM39 were treated (+) or not (-) with cisplatin (50 µM, 24 h). The presence of RBM39 on the *COASY* pre-mRNA was assessed by RNA immunoprecipitation using either control IgG or anti-Flag antibodies. *GAPDH* was used as a negative control. Results are average of three replicates and expressed as mean ± SD, relative to levels of the mRNAs in inputs. Statistical significance was calculated by One-Way ANOVA (****P<0.0001). (**I**) Same as in (E) but MCF-7 cells were depleted from c-Jun by siRNA 24 h prior to the addition of cisplatin.

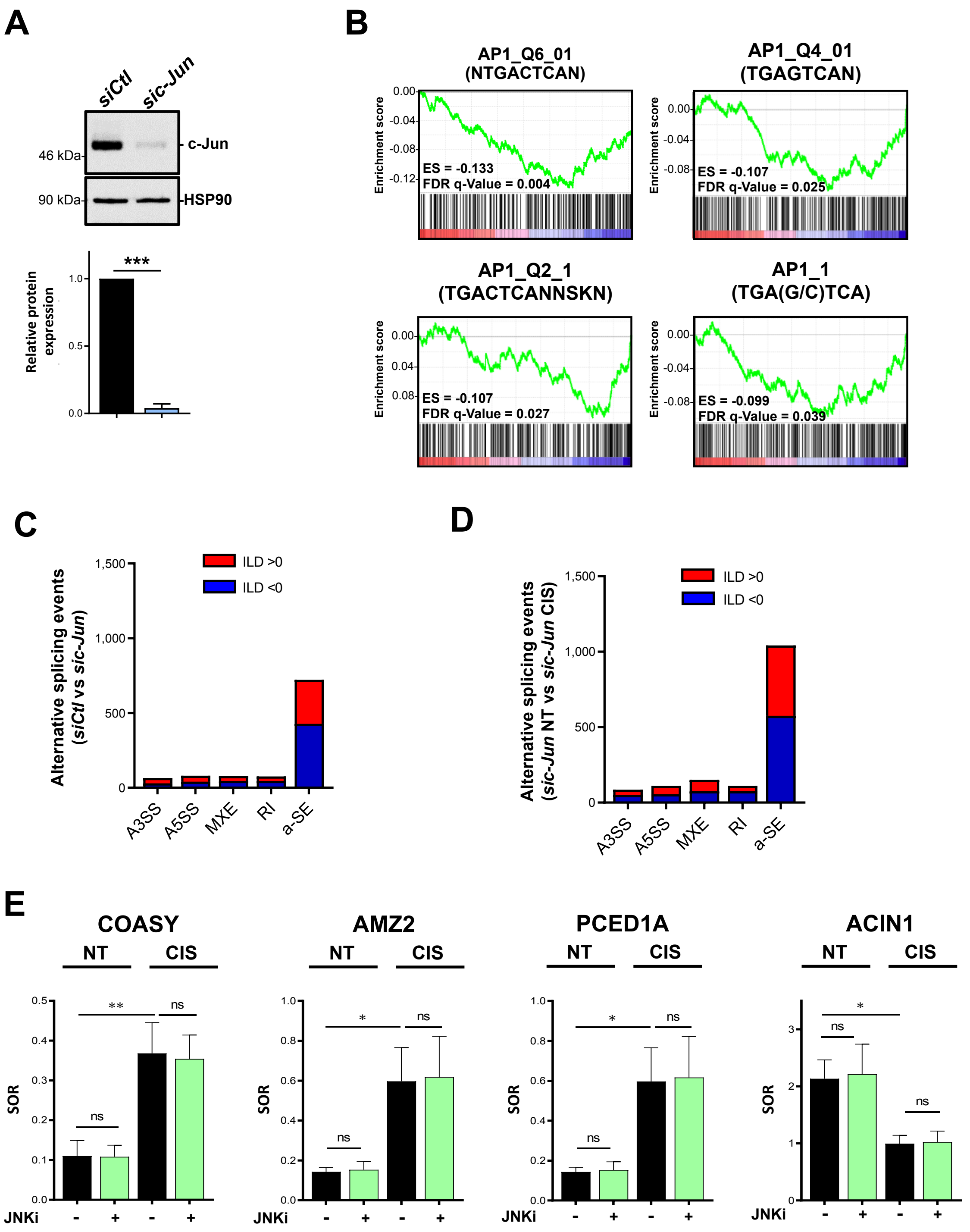

Supplementary Figure 6

Supplementary Figure S6

(**A**) Top: Western blotting of c-Jun following transfection with control (*siCtl*) or a *c-Jun*-specific siRNA (*sic-Jun#1*) in MCF-7 cells used for the genome-wide transcription analysis. HSP90 was used as a loading control. Bottom: Quantification of the RBM39/HSP90 ratio was performed by signal

densitometry analysis and is expressed relative to the *siCtl* condition. Histograms represent means ± SD from three independent experiments. Statistical significance was calculated by Student's *t*-test (****P*<0.001). (**B**) Top ES scores from GSEA against the "C3 transcription factors set" associated with mRNA abundance changes following c-Jun knockdown in MCF-7 cells. (**C**) Numbers and types of alternatively spliced (AS) events after c-Jun knockdown in MCF-7 cells (FDR < 0.05 and |ILD| ≥ 10%, and TPM ≥1 in at least one experimental condition). A3SS: alternative 3' splice site, A5SS: alternative 5' splice site, MXE: mutually exclusive exon (MXE), RI: retained intron and a-SE: alternatively spliced exon. Red and blue bars respectively correspond to AS events with positive and negative ILD. A positive ILD is observed when the inclusion level is higher in control than in cisplatin-treated condition. (**D**) Numbers and types of alternatively spliced (AS) events induced by cisplatin in c-Jun knocked down MCF-7 cells (FDR < 0.05 and |ILD| ≥ 10%, and TPM ≥1 in at least one experimental condition). Red and blue bars respectively correspond to AS events with positive and negative ILD. (**E**) SOR were quantified by RT-PCR for representative Cisplatin-/RBM39-dependent a-SE [exclusion of E4-5 in *COASY*, (n=5); exclusion of E3 in *AMZ2*, (n=3); exclusion of E7 in *PCDE1A* (n=3) and exclusion of E4 in *ACIN1* (n=3)] in MCF-7 cells treated (CIS) or not (NT) with cisplatin (50 µM, 24 h) together (+) or not (-) with a JNK inhibitor (JNKi). Histograms represent means ± SD of SOR quantified from three or five independent experiments as indicated. Statistical significance was calculated by Two-Way ANOVA (ns: not significant, **P*<0.05, ***P*<0.01).
